# Estimating the Association Between Exposome and Psychosis as Well as General Psychopathology: Results From the ABCD Study

**DOI:** 10.1016/j.bpsgos.2022.05.005

**Published:** 2022-06-01

**Authors:** Lotta-Katrin Pries, Tyler M. Moore, Elina Visoki, Ingrid Sotelo, Ran Barzilay, Sinan Guloksuz

**Affiliations:** a Department of Psychiatry and Neuropsychology, School for Mental Health and Neuroscience, Maastricht University Medical Center, Maastricht, the Netherlands; b Department of Psychiatry, Perelman School of Medicine, University of Pennsylvania, Philadelphia, Pennsylvania; c Lifespan Brain Institute, Children’s Hospital of Philadelphia (CHOP), Philadelphia, Pennsylvania; d Department of Child and Adolescent Psychiatry and Behavioral Science, Children’s Hospital of Philadelphia (CHOP), Philadelphia, Pennsylvania; e Penn Medicine, Philadelphia, Pennsylvania; f Department of Psychiatry, Yale University School of Medicine, New Haven, Connecticut

**Keywords:** Adolescents, Adversity, Environment, Exposome, p-factor, Psychopathology, Psychosis

## Abstract

**Background:**

The exposome comprises all nongenetic factors an individual is exposed to across their lifespan. Research suggests that exposomic vulnerability for schizophrenia is associated not only with psychosis but also, to a degree, with general psychopathology. Here, we investigated to what degree exposome factors are associated with psychosis and general psychopathology.

**Methods:**

Data were retrieved from the 1-year follow-up assessment of a large U.S. adolescent sample (*n* = 11,235), the Adolescent Brain Cognitive Development (ABCD) Study. Iterative factor analyses of environmental exposures (*n* = 798) allowed calculation of 6 exposome factors: household adversity, neighborhood environment, day-to-day experiences, state-level environment, family values, pregnancy/birth complications. Bifactor modeling of clinical symptoms (*n* = 93) allowed calculation of a general psychopathology factor (p-factor) and 6 subdomains, including a psychosis subdomain. We applied linear regression analyses to estimate the association of exposome factors with the p-factor and psychosis subdomain, respectively.

**Results:**

Individual analyses showed that 5 exposome factors were significantly associated with the p-factor after multiple-comparison correction. In the mutually adjusted model, all exposome factors were significantly associated with the p-factor. Psychosis was particularly associated with 3 exposome factors, with the mutually adjusted model yielding the following results: household adversity (β = 0.04, 95% CI, 0.01 to 0.07), day-to-day experiences (β = 0.10, 95% CI, 0.08 to 0.12), and pregnancy/birth complications (β = 0.03, 95% CI, 0.01 to 0.05).

**Conclusions:**

Our findings demonstrate that multifaceted environmental background is associated with mental disorders. Psychosis was particularly associated with prenatal, perinatal, and childhood (household and school) adversities, although these exposome domains were also associated with psychopathology. The exposome approach can help understand neurodevelopmental psychopathology.

Psychosis spectrum disorder (PSD) has a complex, multifaceted, interconnected environmental etiology including exposures from different levels (e.g., family-, household-, neighborhood-, and state-level) (1,2). Evidence highlights the role of various environmental exposures such as pre- and perinatal complications, childhood adversities (e.g., abuse, neglect, nonintentional adversities), minority status, cannabis use, and urbanicity (3,4). Research has shown that adverse experiences at different neurodevelopmental sensitive time points (5, 6, 7, 8) such as pregnancy, birth, and childhood (9, 10, 11) have great impact on mental health. Furthermore, not only significant life events but also day-to-day experiences play an important role (1,12, 13, 14). The environment in which a child grows up, including factors such as family, neighborhood, state, or country, can influence their development and psychopathology (1,2,15,16). Furthermore, environmental exposures do not occur in isolation. Exposures are correlated, and they interact with each other (17). For instance, childhood adversity is associated with revictimization (e.g., repeated exposure to sexual assault) (18) and cannabis use (19). There is some evidence that the association between urbanicity and psychosis might be via an indirect path through correlates such as cannabis use, social adversity, exclusion, discrimination (20), and air pollution (21). Studies also suggest a dose–response relationship between exposures and psychopathological outcomes, with an increasing number (or severity) of exposures being associated with increasingly poorer outcomes (22, 23, 24, 25).

This interdependent nature of the environmental etiology of psychosis is not fully understood, which might be (in part) due to the fact that most epidemiological evidence stems from one-exposure-one-outcome approaches. Research has often investigated the association of an isolated exposure, such as childhood adversity, with a particular mental health outcome, such as psychosis. However, this approach does not capture the exposome (26)—the interconnected network of nongenetic exposures an individual is exposed to across their lifetime. The exposome entails exposures from internal (e.g., inflammation) as well as external (e.g., chemical, lifestyle, psychosocial) domains (26). Recently, approaches (such as the identification of individual correlates, mechanisms, and exposome domains) have been applied to uncover exposomic vulnerability for child development by using large numbers of variables (2,27,28). Furthermore, guided by the exposome framework, we previously applied predictive modeling to calculate environmental vulnerability for schizophrenia to estimate an aggregate weighted score (exposome score for schizophrenia) that takes into account the specific loading of individual exposures on schizophrenia, as well as their interdependencies (i.e., correlations) (22). This line of research shows that models that capture the correlation between exposures result in better prediction of psychosis risk than approaches that treat exposures as independent entities (22,29).

Another disadvantage of the one-exposure-one-outcome approach is that it focuses on individual outcomes, thereby ignoring the trans-syndromal nature of psychopathology and nonspecific pluripotent effects of environmental exposures (23,30, 31, 32). Studies that apply the exposome score for schizophrenia show that the exposomic vulnerability for schizophrenia is associated not only with PSD but also with general mental health problems and functioning (12,29,33,34). This finding should not be surprising, given the fact that psychosis expression is on a continuum and coincides with other symptom dimensions (25,35, 36, 37, 38, 39, 40, 41). Furthermore, psychosis expression is often preceded by a nonspecific prodrome with mixed psychopathology domains (42, 43, 44).

In this regard, developmental windows including pregnancy, early childhood, and adolescence may provide important periods of vulnerability (6, 7, 8). The onset of PSD commonly occurs in early adulthood, with deviance from typical neurodevelopmental trajectory taking place in adolescence (8). Psychosis expression is prevalent in young individuals, with around 17% of children (ages 9–12 years) from the general population reporting psychotic experiences (45). The persistence and worsening of psychosis phenotypes is related to impairment, other psychopathology, and genetic as well as environmental vulnerability (43,46, 47, 48, 49, 50). Early psychosis expression may signal an early stage of psychopathology in young individuals, accompanied by a heterogeneous and nonspecific manifestation of clinical symptoms (35,44,45,51, 52, 53).

In this regard, attempting to capture the common liability to different symptom domains, researchers recently investigate the latent p-factor measuring general psychopathology (54). To further investigate the relationship between environment and general psychopathology, we recently took advantage of the large U.S. Adolescent Brain Cognitive Development (ABCD) Study that included comprehensive evaluation of environment. Through an iterative process of repeated factor analyses, we reduced dimensionality of environment from hundreds of exposures to 6 environmental subdomains (i.e., household adversity, neighborhood environment, day-to-day experiences, state-level environment, family values, pregnancy/birth complications).

In our previous work, we estimated a general exposome factor that represents the shared latent construct of all environmental exposures (2) and tested the association of this factor with general psychopathology (p-factor), as well as with general measures of physical health.

The question remains to what degree the individual exposome subdomains explain variance in different dimensions of psychopathology, especially psychosis, when the effect of other multidimensional psychopathology is taken into account. Addressing this question meets the challenge of identifying factors that may be specifically relevant for psychosis phenotypes within general neurodevelopmental psychopathology. The current study builds upon our experience in the previous analysis (2) but aims to understand the specific contribution of environmental subdomains to explain variance in psychosis, over and above their contribution to explaining variance in overall psychopathology. Therefore, the current study leverages a large general population cohort of children and adolescents (the ABCD Study) to investigate the associations of exposome factors with latent dimensions of general psychopathology (i.e., p-factor) and the psychosis domain (sub-/specific factor).

## Methods and Materials

### Participants

Data were retrieved from a large diverse adolescent sample (*N* = 11,878) collected in 21 sites in the United States: the ABCD Study. With the exception of the neuroimaging sites, the ABCD Study generally applied a multistage probability sampling design including a stratified random sample of schools to ensure representativeness of the U.S. children population with an age range of 9 to 10 years at baseline (55). The catchment area encompasses over 20% of the entire U.S. population in this age group. Following a previous report (2), the current study included 1-year follow-up data (*n* = 11,235). Participants provided assent, and parents/caregivers provided signed informed consent. The ABCD Study protocol was approved by the University of California, San Diego Institutional Review Board and was exempted from a full review by the University of Pennsylvania Institutional Review Board (2).

### Measurements

#### Estimating Exposome Factors

To generate exposome factors, we identified 798 variables that capture environmental exposures at multiple levels of analysis including family-, household-, school-, extracurricular-, neighborhood-, and state-level, as well as prenatal exposures. We included measures based on both youth and parent report, as well as the geocoded address (56). We did not include genetic data as we specifically focused on environmental exposures in this project. In addition, we did not include imaging or neurocognitive data. Imaging procedures and the comprehensive ABCD Study neurocognitive assessment were not conducted in the ABCD Study time point used in the current exposome analysis (i.e., 1-year follow-up). We conducted iterative exploratory factor analyses to reduce dimensionality to 6 exposome factors: household adversity, neighborhood environment, day-to-day experiences, state-level environment, family values, and pregnancy/birth complications [see the Supplement and Table S1, and previous report (2) for the details of the procedure].

Thereafter, we fit a correlated-traits constrained factor model (57) from which we calculated exposome factor scores for each participant. Figure S1 provides an overview of exposures with the highest loadings on each of the exposome factors in the correlated-traits exposome model.

#### Estimating General Psychopathology and Psychosis Expression

Similar to previous work in another youth dataset (58) and in the ABCD Study (2), we used clinical items (*n* = 93, including youth self- or caregiver-reported mental health items) to estimate a single dimension of psychopathology (i.e., p-factor) through bifactor modeling. An overview on the scales can be found in the Table S2 and a previous report (2). The bifactor model was used to reduce the dimensionality from the 93 variables to identify (and calculate) the p-factor and to extract 6 subdomains (i.e., psychosis, suicidality, externalizing symptoms, mania, self-reported symptoms, and positive affect). Of note, a major strength of the bifactor model is that subdomains are estimated while taking into account the general psychopathology (p-factor). Therefore, each subdomain covers the weights specific to that particular factor while controlling for other psychopathology. An overview of mental health traits within the general psychopathology bifactor model can be found in a previous publication (2).

For the current study, we were specifically interested in the p-factor and the psychosis subdomain. The psychosis subdomain consisted of the 21 (yes/no) items of the Prodromal Psychosis Scale measuring psychosis spectrum in the ABCD Study (59).

### Statistical Analyses

We used Mplus version 8.4 (60) for factor analyses and SPSS statistical package version 26.0 (IBM Corp.) to test the association between exposome factors and p-factor as well as the psychosis subdomain. First, we analyzed the association between each exposome factor (i.e., household adversity, neighborhood environment, day-to-day experiences, state-level environment, family values, pregnancy/birth complications) and the p-factor in 6 independent analyses. More specifically, we applied 6 linear regression analyses with each individual exposome factor as the independent variable and the p-factor as the dependent variable (Bonferroni-corrected *p* < .008). Following this, we applied a linear regression analysis testing the association between all exposome factors and the p-factor within 1 statistical model (*p* < .05). In this mutually adjusted model, we addressed interdependency among exposome factors through regressing the factors out of each other. We reiterated this analytic approach for the psychosis subdomain as the dependent variable. For the regression analyses, we applied listwise deletion for missing data, which excluded 1019 participants (9.1% of the full sample of *n* = 11,235) (Table S3). We retrieved standardized coefficients. Similar to our previous investigation using data from the ABCD Study, all analyses were adjusted for age, sex, parent education, household income, race (Asian, Black, Other, White), and Hispanic ethnicity.

We also tested the association between the exposome factors and the Prodromal Psychosis Scale severity score to show the difference between the correlated-traits and bifactor measures of psychosis. Furthermore, we conducted several sensitivity analyses to test the robustness of our findings. We analyzed the associations after imputing missing values in the demographic variables, and we applied multilevel mixed models that took into account the clustering of family and site. For details, see the Supplement.

## Results

**Figure 1:**
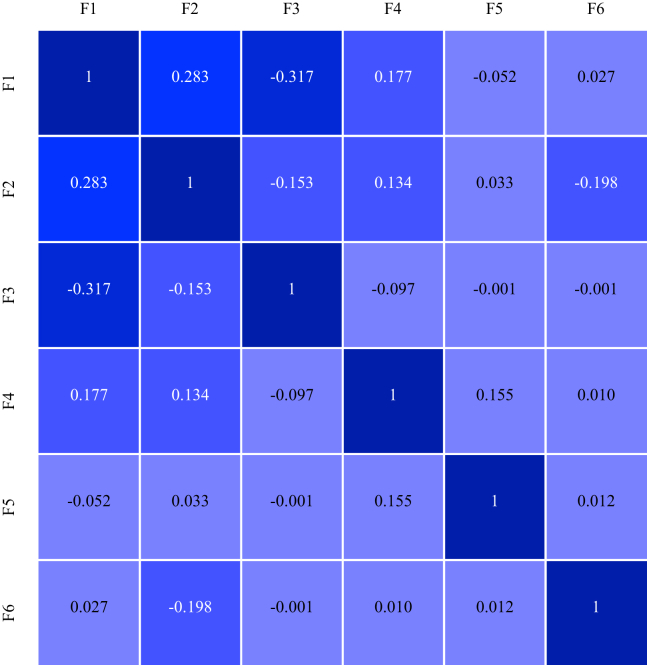
Correlation matrix depicting interfactor correlations among exposome factor (F) scores. A correlation matrix displays interfactor correlations between the 6 correlated exposome factors. Factor 1 comprises variables most related to household adversity. Factor 2 comprises variables most related to neighborhood environment. Factor 3 comprises variables most related to youth-reported day-to-day experiences. Factor 4 comprises variables most related to state-level environment. Factor 5 comprises variables most related to family values. Factor 6 includes variables most related to pregnancy and birth complications.

An overview of demographic variables at the 1-year follow-up assessment is shown in Table 1 (*n* = 11,235; mean age in years = 10.93, 52% male). Table S4 and Figure S1 show results from the correlated-traits factor analysis. Figure 1 shows the interfactor correlations among exposome factors. Figure S1 indicates that among the exposures with the highest loadings on each of the exposome factors, several exposures showed secondary cross loading.

**Table 1:** Demographics of ABCD Study 1-Year Follow-up Assessment

Demographic Variables	*n* (%) or Mean (SD)
Sex, Female	5356 (47.7%)
Age, Years	10.93 (0.64)
Race	
Asian	723 (6.4%)
Black	2269 (20.2%)
Native American	386 (3.4%)
Native Hawaiian/Pacific Islander	70 (0.6%)
White	8453 (75.2%)
Ethnicity, Hispanic	2226 (20.1%)
Household Income	
<$5000	347 (3.3%)
$5000–$11,999	336 (3.2%)
$12,000–$15,999	241 (2.3%)
$16,000–$24,999	446 (4.3%)
$25,000–$34,999	604 (5.8%)
$35,000–$49,999	838 (8.1%)
$50,000–$74,999	1363 (13.2%)
$75,000–$99,999	1460 (14.1%)
$100,000–$199,999	3371 (32.5%)
>$200,000	1358 (13.1%)
Parent Education, Years	16.51 (2.63)

Basic demographic information is presented for the 11,235 participants that make up the study population, including age, sex, race, ethnicity, household income, and parents’ education.

ABCD, Adolescent Brain Cognitive Development.

### Associations Between Exposome Factor Scores and General p-Factor

The investigation of the associations between exposome factors and the p-factor applying 6 independent analyses showed that 5 exposome factors were associated with the p-factor, controlling for covariates (Table 2). Family values (β = −0.05, 95% CI, −0.07 to −0.03, *p* < .001) were associated with lower psychopathology, whereas the other exposome factors were associated with greater psychopathology: household adversity (β = 0.31, 95% CI, 0.28 to 0.33, *p* < .001), day-to-day experiences (β = 0.62, 95% CI, 0.61 to 0.64, *p* < .001), state-level environment (β = 0.07, 95% CI, 0.05 to –0.09, *p* < .001), and pregnancy/birth complications (β = 0.04, 95% CI 0.02 to –0.06, *p* < .001). The association between neighborhood environment (β = 0.03, 95% CI, 0.00 to –0.05, *p* = .045) and psychopathology was not statistically significant after Bonferroni correction (*p* < .008).

**Table 2:** Associations of Exposome Factor Scores With Psychosis Subdomain and General p-Factor

	Psychosis Subdomain			General p-Factor		
Exposome Factor	β	95% CI	*p*	β	95% CI	*p*
Household Adversity	0.028	0.006 to 0.050	.014	0.305	0.283 to 0.327	<.001
Neighborhood Environment	0.017	−0.009 to 0.043	.190	0.026	0.000 to 0.052	.045
Day-to-Day Experiences	0.094	0.074 to 0.114	<.001	0.622	0.606 to 0.638	<.001
State-Level Environment	−0.021	−0.041 to −0.001	.041	0.071	0.051 to 0.091	<.001
Family Values	−0.006	−0.026 to 0.014	.571	−0.050	−0.070 to −0.030	<.001
Pregnancy/Birth Complications	0.032	0.012 to 0.052	.001	0.036	0.016 to 0.056	<.001

Each exposome factor was tested in a separate model (6 models for each dependent variable). Models covaried for age, sex, race, ethnicity, household income, and parental education.

**Figure 2:**
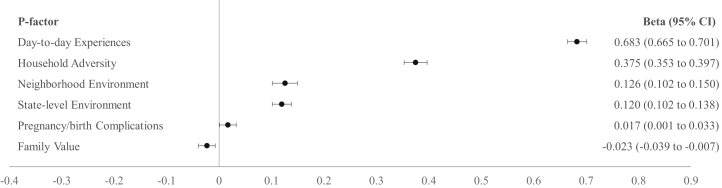
Associations of exposome factor scores with the p-factor. All exposome factors are included in a single model. To address collinearity among exposome factors, they were regressed out of each other. Models covaried for age, sex, race, ethnicity, household income, and parental education. For visualization, results are sorted by the highest to lowest βs (standardized coefficients).

The mutually adjusted analysis showed that all exposome factors were statistically significantly associated with the p-factor (Figure 2). The final analysis including all exposome factors and covariates explained 40.1% of the variance in the p-factor (adjusted *R*^2^ = 0.401).

### Associations Between Exposome Factor Scores and Psychosis Subdomain

The investigation of the associations between exposome factors and the psychosis subdomain applying 6 independent analyses showed that day-to-day experiences (β = 0.09, 95% CI, 0.07 to –0.11, *p* < .001) and pregnancy/birth complications (β = 0.03, 95% CI 0.01 to –0.05, *p* = .001) were associated with higher values on the psychosis subdomain. The associations of household adversity (β = 0.03, 95% CI, 0.01 to –0.05, *p* = .014) and state-level environment (β = −0.02, 95% CI, −0.04 to −0.00, *p* = .041) were not statistically significant after Bonferroni correction (*p* < .008). No other statistically significant associations were found (Table 2).

**Figure 3:**
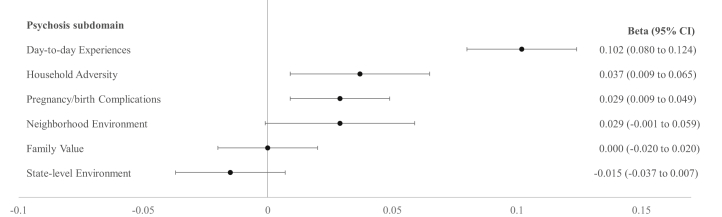
Associations of exposome factor scores with the psychosis subdomain. All exposome factors are included in a single model. To address collinearity among exposome factors, they were regressed out of each other. Models covaried for age, sex, race, ethnicity, household income, and parental education. For visualization, results are sorted by the highest to lowest βs (standardized coefficients).

The mutually adjusted analysis showed that household adversity (β = 0.04, 95% CI, 0.01 to 0.07, *p* = .009), day-to-day experiences (β = 0.10, 95% CI, 0.08 to –0.12, *p* < .001), and pregnancy/birth complications (β = 0.03, 95% CI, 0.01 to –0.05, *p* = .005) were statistically significantly associated with the psychosis subdomain (Figure 3). The association between neighborhood environment and psychosis was trend significant (β = 0.03, 95% CI, −0.00 to –0.06, *p* = .058). No other statistically significant associations were found. The final analysis including all exposome factors and covariates explained 4.8% of the variance in the psychosis subdomain (adjusted *R*^2^ = 0.048). For comparison, the analyses testing the association between the exposome factors and the severity score of the Prodromal Psychosis Scale can be found in the Supplement.

### Sensitivity Analyses

The sensitivity analyses provided support for the robustness of the findings. Results from imputed data converged with those from unimputed data (Tables S7, S8). The analyses adjusting for site and family were also similar. Exposome factors were statistically significantly associated with the p-factor in the independent analyses and the mutually adjusted analysis (Table S9). Household adversity, neighborhood environment, and day-to-day experiences were significantly associated with psychosis factor in the independent analyses and the mutually adjusted analysis (Table S10). Furthermore, pregnancy/birth complications were associated with the psychosis factor in the mutually adjusted analysis (β = −0.04, 95% CI, −0.06 to −0.01, *p* = .003).

## Discussion

We investigated the association of 6 exposome factors (i.e., household adversity, neighborhood environment, day-to-day experiences, state-level environment, family values, pregnancy/birth complications) with a latent general psychopathology p-factor as well as the psychosis subdomain. Our findings illustrate that all exposome factors were associated with general psychopathology in a single model that accounts for collinearity among all exposome factors (i.e., mutually adjusted model). Furthermore, we found that household adversity, day-to-day experiences, and pregnancy/birth complications were specifically associated with the psychosis subdomain. The finding that multiple exposome factors were associated with the p-factor and psychosis subdomain is in accordance with research showing the multifactorial etiology of neurodevelopmental psychopathology (28,61).

The mutually adjusted analyses revealed that only 3 exposome factors were associated with the psychosis subdomain, while all exposome factors were associated with the p-factor. Furthermore, standardized coefficients were generally higher for the associations with the p-factor than for the association with the psychosis subdomain. Notably, the exposome factors explained >40% of the p-factor, compared with 4.8% of the psychosis subdomain. This can be expected as the p-factor captures the broad range of multidimensional psychopathology that is commonly affected by environmental etiology. Combined with converging evidence (12,22,28,29), this study shows that the exposome approach helps to provide a better understanding of the complex network of environmental vulnerability for mental health than the study of individual environmental factors in isolation.

The exposome factor that was associated with the p-factor and psychosis subdomain with the largest standardized coefficient was the day-to-day experiences (that includes family relationships, school environment, and interpersonal stressors such as discrimination). This finding might be related to the commonly observed effect of proximity of stressors on mental health measures (12, 13, 14). Research shows that (day-to-day) stressors that are in close proximity (e.g., temporal or personal) have a more severe or direct impact on the outcome than distal stressors (1,12, 13, 14). Furthermore, the items constituting day-to-day experiences were based on youth reports. Therefore, the experiences may have been more personal, acute stressors and provided more accurate assessment of the children’s situation. These findings are in agreement with the literature on the association of discrimination, social experiences, and parent–child relationships with psychopathology and psychosis (1). Future studies investigating the temporal relation between the exposome factors and mental health outcomes are warranted to test the temporal association of this exposure-outcome relationship.

In addition to day-to-day experiences, both household adversity and pregnancy/birth complications were specifically relevant for psychosis as well as general psychopathology. Both of these domains were previously associated with psychosis phenotypes as well as other domains of mental health (4,9,10,62,63). Household adversity exposome factor captures aspects of childhood adversity (e.g., severe poverty, physical violence in the household), which is one of the most studied risk factors for psychopathology (62,63). Our findings are in accordance with other studies that use baseline ABCD Study data showing that environmental exposures such as prenatal exposure to tobacco or marijuana (64) and childhood adversities (11) are associated with psychosis and general psychopathology. Furthermore, the association between parent-reported adverse childhood experiences and psychosis expression appears to be above and beyond other shared correlates (e.g., everyday stress, depression, and anxiety symptoms) (11). Research likewise shows that pre- and perinatal complications are associated with PSD and psychopathology (9,10). However, it is noteworthy to mention that information about pregnancy complications are less often collected and difficult to consistently assess in large cohort studies without medical records, as retrospective assessment shows low reliability. Counterintuitively, the sensitivity analyses adjusting for family and site indicated a negative association between pregnancy/birth complications and the psychosis factor in the mutually adjusted model. This finding indicates that there might be site-specific differences that might distort the associations. It is possible that some sites may have better access to peri- and prenatal medical care than other sites, thereby resulting in differences of reported pregnancy/birth complications. Therefore, further analyses are needed to understand possible site-specific effects. Our findings will hopefully encourage researchers to include assessments of early pre- and perinatal adversities in deeply phenotyped cohort studies. Our findings also highlight the specific role of adversity during neurodevelopmentally sensitive time periods.

State-level environment (e.g., laws in specific states and indicators of bias against sexual orientation, sexism, or racism) was significantly associated with increased psychopathology but not with psychosis. This exposome factor entails different proxy items for known (e.g., legalization of medical marijuana as a possible proxy for higher rates of cannabis use) and possibly unknown exposures that form a complex network. It is difficult to evaluate which correlates might have had an impact on the individual level and subsequently on the manifestation of psychopathology. State-level environment might have entailed proxies of several factors such as cannabis use, social adversity, exclusion, and discrimination that are consistently associated with psychopathology as well as psychosis (20,65, 66, 67). However, our results suggest that the exposures in state-level environment may not be specifically associated with psychosis but generally with psychopathology. Furthermore, the items within this factor might have tapped into other domains within the exposome (e.g., neighborhood environment). Future research is needed to understand the effect of the individual components of this exposome factor on psychopathology.

The current study confirms evidence for an association between neighborhood environment and psychopathology (1), which was shown specifically in youth (32). This is in line with a previous study that used data from the ABCD Study. The study showed that environmental variables retrieved through geocoded address (neighborhood environment) were associated with psychopathology (61). Although neighborhood environment was not statistically significantly associated with psychosis in the main analyses, it was significant in the sensitivity analyses adjusting for family and site. The latter result is supported by previous findings suggesting an association of the geocoded environmental risk factors and psychosis (61). However, neighborhood environment might already include environmental background that is also captured by sites. Therefore, future research needs to carefully assess site-specific effects.

Family values were associated with reduced psychopathology. This factor entails several items related to families’ attitudes toward substance abuse (e.g., family rules for using marijuana, smoking cigarettes, and drinking alcohol). On the one hand, the attitude might have impacted children’s attitude as well as behavior toward substances, and eventually psychopathology. On the other hand, parental abuse of substances is commonly associated with health issues in children (68). Items tapping into religiosity and family support may underline the possible protective effects of social networks and social capital (1). Given potential intervention opportunities, further research on the causal effect of this exposome factor on psychopathology is needed.

Although studies indicate that aggregated “risk” scores for schizophrenia are associated with PSD (22,69), with higher risk and higher explained variance compared with other phenotypes, they appear to be pluripotent rather than being only specific to psychosis. Similar to genetic vulnerability, environmental vulnerability for schizophrenia is associated with an extended psychosis phenotype as well as broad mental health and physical health problems (12,29). The current study adds to previous literature by highlighting potential target exposome domains that might be specifically important for psychosis expression as well as psychopathology. In this regard, it is important to note that psychosis co-occurs with other multidimensional psychopathology (11,25,35) and physical complaints (70) and is bidirectionally associated with psychopathology (44). A previous ABCD Study analysis showed that psychosis expression mediated the association between childhood adversity and internalizing symptoms as well as suicidality (11). Furthermore, different factors such as use of mental health services, drop in grades, other symptoms, adversities, and suicidality were associated with more sustained versus transient psychosis expression (50). Given that the magnitude of psychosis admixture depends on environmental exposures (25,71), future studies need to further evaluate to what degree the exposome factors contribute to the development of psychosis admixture especially when applying prospective approaches. Furthermore, to test the specificity of the exposome factors, researchers should also test the associations with other psychopathology, cognition, and behavioral domains.

In accordance with the diathesis-stress model (72), early-life environmental and genetic vulnerability interact with exposures later in life to drive psychopathology (12). Previous works using ABCD Study data indicated that direct and indirect measures of genetic risk for psychosis and mental health were associated with psychosis expression (39,73). Therefore, future research may aim to investigate whether exposome dimensions interact with genetic vulnerability and whether this may be associated with psychosis phenotypes and psychosis admixture. Future studies are needed to understand the effects of the individual exposures and possibly their interactions within the exposome factors.

### Limitations

The current study takes advantage of a large deeply phenotyped adolescent cohort to test the association of exposome factors with psychosis as well as general psychopathology. Nonetheless, several limitations should be mentioned. First, a priori decision-making processes were applied to preselect important correlates. The large number of exposures was substantially reduced according to previous knowledge and common sense. Summary values rather than raw data were included in several instances. Although this approach ensured the selection of important correlates for psychopathology, different approaches might have resulted in different outcomes. Furthermore, an extended coverage of other correlates such as indoor air pollutants (74) or persistent organic pollutants in breast milk (75) would have provided a more complete set of exposures. However, inclusion of variables was limited to the availability in the ABCD Study. Second, the analyses were adjusted for several important demographic covariates. However, collider bias may occur if both the exposure and the outcomes causally impact the covariates (e.g., both exposome and psychosis may influence household income). Longitudinal analyses are therefore needed. Third, some variables (such as family psychiatric history) capture not only environmental impact (e.g., through parent deprivation, child–parent separation due to hospitalization) (76) but also genetic vulnerability (77). Future studies that control for direct measures of genetic vulnerability (e.g., polygenic scores) may help disentangle genetic and environmental components to a degree. Fourth, the study provided valuable information on the cross-sectional association between exposome factors and psychosis expression as well as psychopathology in the 1-year follow-up snapshot of the ABCD Study. However, not all individuals who have exposomic vulnerability may develop distinct mental health problems later in life. Early expression of psychosis might follow a heterotypic course and evolve into other nonpsychotic psychiatric diagnoses. Of note, some exposome factors, such as day-to-day experiences, were mainly based on youth reports, thereby making causal inferences especially difficult, as children with mental health problems may have been more prone to report negative (day-to-day) experiences. To infer causality and investigate exposome effects on trajectories, future cohort studies with longer follow-up are needed. Fifth, instead of using a discovery and replication sample, the exposome and clinical models were estimated in one discovery cohort. However, our aim was not to test/confirm a specific theoretical structure [as in (78)] but rather to estimate optimal empirically derived scores in the ABCD Study cohort for multiple downstream analyses. Future longitudinal approaches using the ABCD Study cohort will help cross-validate these measurement models.

### Conclusions

In combination with previous research, our findings underline the multifaceted etiology of youth mental health. Psychosis expression was especially associated with prenatal, perinatal, and childhood adversities. However, these factors were also associated with general psychopathology. These findings underscore the important role of early environmental adversities during neurodevelopmentally sensitive windows. The exposome approach can help understand the development of neurodevelopmental psychopathology.

## Supplementary Material

Supplementary Material

Key Resources Table
