# Supplementary material for: Estimating the Association Between Exposome and Psychosis as Well as General Psychopathology: Results From the ABCD Study
Source: Biol Psychiatry Glob Open Sci. 2022 Jun 1;2(3):283–91. doi: 10.1016/j.bpsgos.2022.05.005 (PMC9616253; doi:10.1016/j.bpsgos.2022.05.005)
Supplement: Supplementary Material [file mmc1.pdf]

Estimating the Association Between Exposome and Psychosis as Well as General Psychopathology: Results From the ABCD Study

Supplemental Information

Table of Contents

Supplementary Material **2**

Calculation of correlated exposome factor scores 2

Sensitivity analyses 3

Differences between the psychosis factor and the severity score of the ABCD Prodromal Psychosis Scale 3

Supplementary Figure 1. Correlated traits exposome model **5**

Supplementary Table 1 Full set of exposome measures included in ABCD Study analysis . **6**

Supplementary Table 2 Overview of ABCD instruments assessing mental health items **90**

Supplementary Table 3 Missing data **91**

Supplementary Table 4. Correlated traits factor analysis of optimized collection of exposome variables **92**

Supplementary Table 5 Associations of exposome factor scores with the PQ-B severity score **96**

Supplementary Table 6 Explained variance (R^2) of the exposome factors and the covariates on the p-factor, psychosis factor, as well as the PQ-B **97**

Supplementary Table 7 Associations of exposome factor scores with general p-factor imputed for missing demographic variables **98**

Supplementary Table 8 Associations of exposome factor scores with psychosis factor imputed for missing demographic variables **99**

Supplementary Table 9 Associations of exposome factor scores with general p-factor adjusted for family and site **100**

Supplementary Table 10 Associations of exposome factor scores with psychosis factor adjusted for family and site **101**

Supplementary References **102**

Supplementary Material

Calculation of correlated exposome factor scores

We started with 798 variables, from which we selected certain ABCD-provided summary variables according to a combination of *a priori* knowledge (e.g. similar decisions had to be made about the American Community Survey in our previous work(1) and common sense, ultimately reducing the variable count to 348. We often chose to use summary scales to represent overarching culture and environment (e.g., Mexican American Cultural Values Scale, family conflict) and indicators of health (e.g., family psychiatric history, dietary habits). We included these in the following analysis, and by using multiple exploratory factor analyses (EFAs), we iteratively reduced the number of variables to 96 variables with minimal redundancy.

Next, we estimated an EFA solution using the "clean" 96-variable dataset obtained from the iterative process described above. A unique aspect of this step was that, because we expected complex structure whereby some cross-loadings would be substantial and meaningful, we used iterated target rotation (ITR)(2, 3) rather than a simple structure rotation like oblimin or promax. Simple structure rotations attempt to get p-1 elements in each row as close to zero as possible (where p = number of factors), but ITR allows salient cross-loadings to be estimated freely. It starts with a simple structure rotation (here, oblimin), uses the resulting pattern matrix to determine not only which item loads where but also which cross-loadings might be non-negligible, and builds a partially-specified target matrix that incorporates cross-loading items(4). Specifically, it uses a user-defined threshold (here, 0.20), sets all elements of the target matrix at 0 for items loading below that threshold, and sets all other (non-negligible) loadings to "unspecified" (indicating they should be estimated freely). The results of this target rotation are then used in the same way as the original simple structure rotation to specify a new target, and the process is repeated. When a new target matrix matches a previous target matrix in the iterative process, the ITR solution has converged.

Lastly, to generate exposome factor scores, we fit a correlated traits model(5) that resulted in calculation of six correlated exposome factor scores.

Sensitivity analyses

To test the robustness of our findings, we conducted two sensitivity analyses. First, we analyzed the associations after imputing for missing values (**Supplementary Table 3**) in the demographic variables using the missForest package(6) in R. We applied single-imputation via random forest which is an iterative process with several steps: 1) all variables except one are used to predict (via a random forest model) the left-out variable. Any missing values in the independent variables are replaced by the mean to ensure non-missing predicted values for all rows of the left-out variable; 2) with one variable imputed, the algorithm moves on to the next variable, imputing (predicting) it using a new random forest model comprising all other variables (including the variable that has just been imputed); 3) the algorithm cycles through all variables and then restarts step #1 above ("cycling back" to the original left-out variable), where missing values in the independent variables no longer need mean-replacement because they were imputed in the previous imputation cycle (steps 1 & 2 above); 4) the algorithm repeats steps 1 through 3 until the imputed values stop changing across imputation cycles.

Second, we applied Mixed Effects Regression with random intercepts for family and site to test the association of exposome factors with the p-factor and the psychosis factor while taking into account the clustering of family and site.

Differences between the psychosis factor and the severity score of the ABCD Prodromal Psychosis Scale

The bifactor psychosis factor score is derived from weights specific to psychosis (after controlling for "p"), while the Prodromal Psychosis Scale (PQ-B) score is "contaminated" by overall psychopathology ("p"). However, according to the literature(7-10), it is not yet clear what bifactor-modeled psychosis actually is. It might be that the p-factor is simply "response propensity" (tendency to answer items in the affirmative), in which case the specific factors are "pure" only insofar as they control for nuisance response propensity. However, it is unlikely to be that simple as discussed(7-10). Therefore, we analyzed the association between the exposome factors and the severity score of the PQ-B to indicate the differences between the correlated-traits and bifactor measures of psychosis.

To analyze the association, we applied six linear regression analyses with each individual exposome factor as the independent variable and the severity score of the PQ-B as the

dependent variable. For the six individual analyses, a Bonferroni corrected P<0.008 was considered statistically significant. Following this, we applied a linear regression analysis testing the association between all exposome factors and the severity score of the PQ-B within one statistical model (with *P*<0.05).

In the six individual analyses, all exposome factors were associated with the severity score of the PQ-B, either significantly (i.e. below the Bonferroni corrected p-threshold) or significant at a trend level (*P*<0.05; **Supplementary Table 5**). Likewise, most exposome factors were also significantly associated with the severity score of the PQ-B in the mutually adjusted analysis. Only family values were not statistically significantly associated with the severity score of the PQ-B in the mutually adjusted analysis **(Supplementay Table 5)**. In contrast, the psychosis factor was specifically associated (*P*<0.008) with day-to-day experiences and pregnancy/birth complications in the six individual analyses as well as with household adversity, day-to-day experiences, and pregnancy/birth complications in the mutually adjusted model (*P*<0.05; **Table 2**). For comparison, **Supplementary Table 6** shows the explained variance for the psychosis factor, p-factor, and the PQ-B in the six independent analyses, respectively. The results highlight the differences between the correlated-traits and bifactor measures of psychosis, and identify the exposome factors that are specifically relevant for the bifactor measures of psychosis controlling for “p”.

Supplementary Figure 1. Correlated traits exposome model

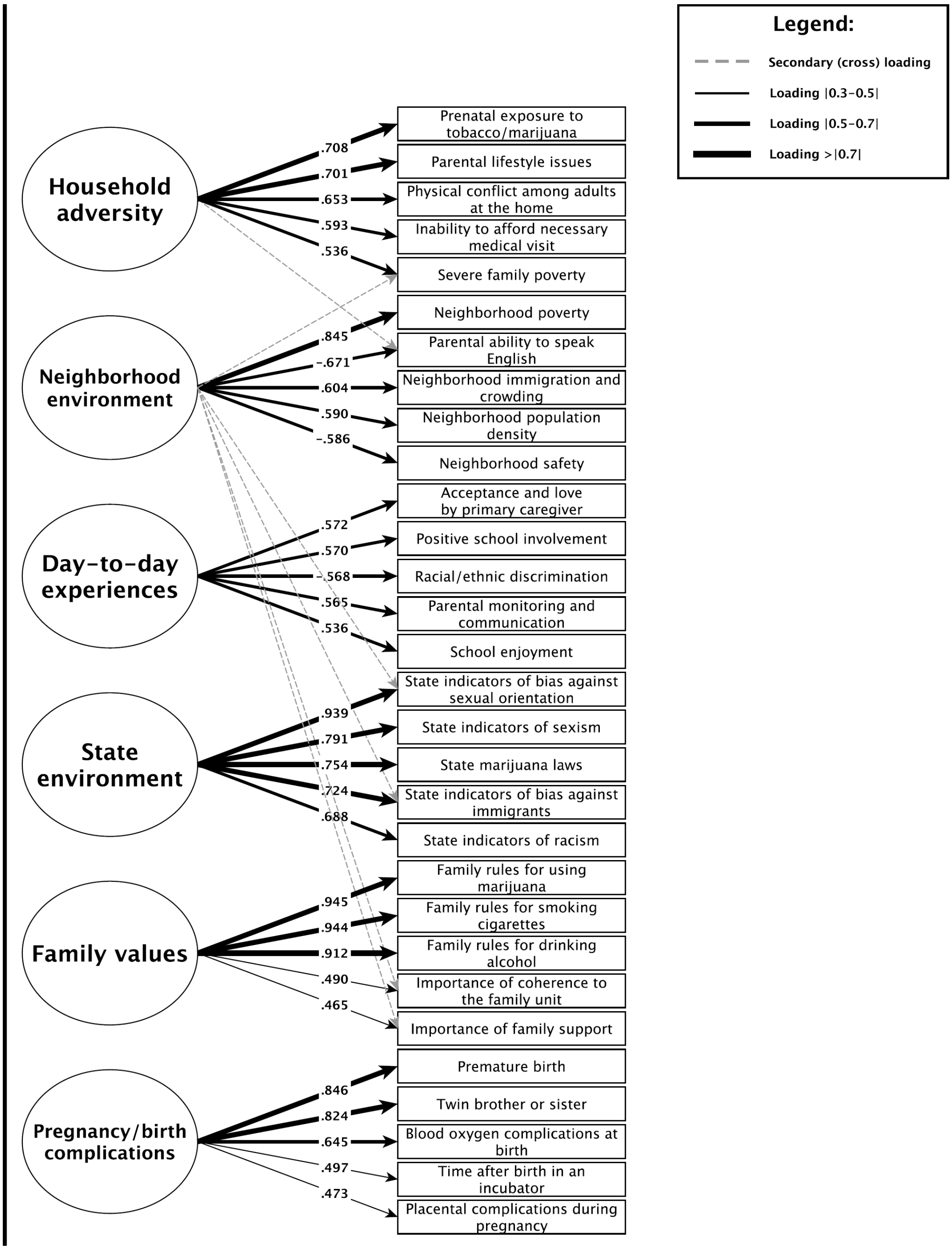

Supplementary Table 1 Full set of exposome measures included in ABCD Study analysis

ABCD instrument	ABCD item code	ABCD item description	ABCD subinstrument code	ABCD subitem code	ABCD subitem description	Reporter	Time point
ABCD Longitudinal Summary Scores Sports Activity (abcd_lsssa01)	sai_ss_dance_nmonth_p_l	Months per year ballet/dance (past year)	abcd_lpsaiq01	sai_p_activities_l___0	Ballet, Dance	Parent	1-year follow-up
				sai_p_dance_nmonth_l	Since we last saw you, about how many months did your child participate in this activity?	Parent	1-year follow-up
	sai_ss_base_nmonth_p_l	Months per year baseball/softball (past year)		sai_p_activities_l___1	Baseball, Softball	Parent	1-year follow-up
				sai_p_base_nmonth_l	Since we last saw you, about how many months did your child participate in this activity?	Parent	1-year follow-up
	sai_ss_basket_nmonth_p_l	Months per year basketball (past year)		sai_p_activities_l___2	Basketball	Parent	1-year follow-up
				sai_p_basket_nmonth_l	Since we last saw you, about how many months did your child participate in this activity?	Parent	1-year follow-up
	sai_ss_climb_nmonth_p_l	Months per year climbing (past year)		sai_p_activities_l___3	Climbing	Parent	1-year follow-up
				sai_p_climb_nmonth_l	Since we last saw you, about how many months did your child participate in this activity?	Parent	1-year follow-up

sai_ss_fhock_nmonth_p_l	Months per year field hockey (past year)	sai_p_activities_l___4	Field hockey	Parent	1-year follow-up
		sai_p_fhock_nmonth_l	Since we last saw you, about how many months did your child participate in this activity?	Parent	1-year follow-up
sai_ss_fball_nmonth_p_l	Months per year football (past year)	sai_p_activities_l___5	Football	Parent	1-year follow-up
		sai_p_fball_nmonth_l	Since we last saw you, about how many months did your child participate in this activity?	Parent	1-year follow-up
sai_ss_gym_nmonth_p_l	Months per year gymnastics (past year)	sai_p_activities_l___6	Gymnastics	Parent	1-year follow-up
		sai_p_gym_nmonth_l	Since we last saw you, about how many months did your child participate in this activity?	Parent	1-year follow-up
sai_ss_ihock_nmonth_p_l	Months per year ice hockey (past year)	sai_p_activities_l___7	Ice Hockey	Parent	1-year follow-up
		sai_p_ihock_nmonth_l	Since we last saw you, about how many months did your child participate in this activity?	Parent	1-year follow-up
sai_ss_polo_nmonth_p_l	Months per year horseback riding/polo (past year)	sai_p_activities_l___8	Horseback Riding, Polo	Parent	1-year follow-up

				sai_p_polo_nmonth_l	Since we last saw you, about how many months did your child participate in this activity?	Parent	1-year follow-up
	sai_ss_iskate_nmonth_p_l	Months per year ice/inline skating (past year)		sai_p_activities_l___9	Ice or Inline Skating	Parent	1-year follow-up
				sai_p_iskate_nmonth_l	Since we last saw you, about how many months did your child participate in this activity?	Parent	1-year follow-up
	sai_ss_m_arts_nmonth_p_l	Months per year martial arts (past year)		sai_p_activities_l___10	Martial Arts	Parent	1-year follow-up
				sai_p_m_arts_nmonth_l	Since we last saw you, about how many months did your child participate in this activity?	Parent	1-year follow-up
ABCD Longitudinal Summary Scores Sports Activity (abcd_lsssa01)	sai_ss_lax_nmonth_p_l	Months per year lacrosse (past year)	abcd_lpsaiq01	sai_p_activities_l___11	Lacrosse	Parent	1-year follow-up
				sai_p_lax_nmonth_l	Since we last saw you, about how many months did your child participate in this activity?	Parent	1-year follow-up
	sai_ss_rugby_nmonth_p_l	Months per year rugby (past year)		sai_p_activities_l___12	Rugby	Parent	1-year follow-up
				sai_p_rugby_nmonth_l	Since we last saw you, about how many months did your child participate in this activity?	Parent	1-year follow-up

sai_ss_skate_nmonth_p_l	Months per year skateboarding (past year)	sai_p_activities_l___13	Skateboarding	Parent	1-year follow-up
		sai_p_skate_nmonth_l	Since we last saw you, about how many months did your child participate in this activity?	Parent	1-year follow-up
sai_ss_sboard_nmonth_p_l	Months per year skiing/snowboarding (past year)	sai_p_activities_l___14	Skiing, Snowboarding	Parent	1-year follow-up
		sai_p_sboard_nmonth_l	Since we last saw you, about how many months did your child participate in this activity?	Parent	1-year follow-up
sai_ss_soc_nmonth_p_l	Months per year soccer (past year)	sai_p_activities_l___15	Soccer	Parent	1-year follow-up
		sai_p_soc_nmonth_l	Since we last saw you, about how many months did your child participate in this activity?	Parent	1-year follow-up
sai_ss_surf_nmonth_p_l	Months per year surfing (past year)	sai_p_activities_l___16	Surfing	Parent	1-year follow-up
		sai_p_surf_nmonth_l	Since we last saw you, about how many months did your child participate in this activity?	Parent	1-year follow-up
sai_ss_wpolo_nmonth_p_l	Months per year swimming/water polo (past year)	sai_p_activities_l___17	Swimming, Water Polo	Parent	1-year follow-up

		sai_p_wpolo_nmonth_l	Since we last saw you, about how many months did your child participate in this activity?	Parent	1-year follow-up
sai_ss_tennis_nmonth_p_l	Months per year tennis (past year)	sai_p_activities_l___18	Tennis	Parent	1-year follow-up
		sai_p_tennis_nmonth_l	Since we last saw you, about how many months did your child participate in this activity?	Parent	1-year follow-up
sai_ss_run_nmonth_p_l	Months per year track/running/cross-country (past year)	sai_p_activities_l___19	Track, Running, Cross-country	Parent	1-year follow-up
		sai_p_run_nmonth_l	Since we last saw you, about how many months did your child participate in this activity?	Parent	1-year follow-up
sai_ss_mma_nmonth_p_l	Months per year wrestling/mixed martial arts (past year)	sai_p_activities_l___20	Wrestling, Mixed Martial Arts	Parent	1-year follow-up
		sai_p_mma_nmonth_l	Since we last saw you, about how many months did your child participate in this activity?	Parent	1-year follow-up
sai_ss_vball_nmonth_p_l	Months per year volleyball (past year)	sai_p_activities_l___21	Volleyball	Parent	1-year follow-up
		sai_p_vball_nmonth_l	Since we last saw you, about how many months did your child participate in this activity?	Parent	1-year follow-up

	sai_ss_yoga_nmonth_p_l	Months per year yoga/tai chi (past year)		sai_p_activities_l___22	Yoga, Tai Chi	Parent	1-year follow-up
				sai_p_yoga_nmonth_l	Since we last saw you, about how many months did your child participate in this activity?	Parent	1-year follow-up
	sai_ss_music_nmonth_p_l	Months per year playing musical instrument (singing/choir/guitar/piano/drums/violin/flute/band/rock band/orchestra) (past year)		sai_p_activities_l___23	Musical Instrument (Singing, Choir, Guitar, Piano, Drums, Violin, Flute, Band, Rock Band, Orchestra)	Parent	1-year follow-up
ABCD Longitudinal Summary Scores Sports Activity (abcd_lsssa01)	sai_ss_music_nmonth_p_l	Months per year playing musical instrument (singing/choir/guitar/piano/drums/violin/flute/band/rock band/orchestra) (past year)	abcd_lpsaiq01	sai_p_music_instr___0	Guitar, Bass Guitar, Ukulele	Parent	1-year follow-up
				sai_p_music_instr___1	Flute, Piccolo, Recorder	Parent	1-year follow-up
				sai_p_music_instr___2	Clarinet, Saxophone, Oboe, Bassoon	Parent	1-year follow-up
				sai_p_music_instr___3	Violin, Viola	Parent	1-year follow-up
				sai_p_music_instr___4	Cello, Bass	Parent	1-year follow-up
				sai_p_music_instr___5	Piano	Parent	1-year follow-up

		sai_p_music_instr___6	Harp	Parent	1-year follow-up
		sai_p_music_instr___7	Drums, Percussion	Parent	1-year follow-up
		sai_p_music_instr___8	Trumpet, Trombone, Horns	Parent	1-year follow-up
		sai_p_music_instr___9	Vocal, Singing	Parent	1-year follow-up
		sai_p_music_instr___10	Electric Keyboard, Organ	Parent	1-year follow-up
		sai_p_music_instr___11	DJ, Electronic Dance Music	Parent	1-year follow-up
		sai_p_music_instr___12	Other	Parent	1-year follow-up
		sai_p_music_nmonth_l	Since we last saw you, about how many months did your child participate in this activity?	Parent	1-year follow-up
sai_ss_art_nmonth_p_l	Months per year drawing/painting/graphic	sai_p_activities_l___24	Drawing, Painting, Graphic Art, Photography, Pottery, Sculpting	Parent	1-year follow-up

	art/photography/pottery/sculpting (past year)	sai_p_art_nmonth_l	Since we last saw you, about how many months did your child participate in this activity?	Parent	1-year follow-up
sai_ss_drama_nmonth_p_l	Months per year drama/theater/acting/film (past year)	sai_p_activities_l___25	Drama, Theater, Acting, Film	Parent	1-year follow-up
		sai_p_drama_nmonth_l	Since we last saw you, about how many months did your child participate in this activity?	Parent	1-year follow-up
sai_ss_crafts_nmonth_p_l	Months per year doing crafts like knitting/building model cars or airplanes (past year)	sai_p_activities_l___26	Crafts like Knitting, Building Model Cars or Airplanes	Parent	1-year follow-up
		sai_p_crafts_nmonth_l	Since we last saw you, about how many months did your child participate in this activity?	Parent	1-year follow-up
sai_ss_chess_nmonth_p_l	Months per year playing competitive games like chess/cards/darts (past year)	sai_p_activities_l___27	Competitive Games like Chess, Cards, or Darts	Parent	1-year follow-up
		sai_p_chess_nmonth_l	Since we last saw you, about how many months did your child participate in this activity?	Parent	1-year follow-up
sai_ss_collect_nmonth_p_l	Months per year participating in hobbies like collecting stamps or coins (past year)	sai_p_activities_l___28	Hobbies like collecting stamps or coins	Parent	1-year follow-up
		sai_p_collect_nmonth_l	Since we last saw you, about how many months did your child participate in this activity?	Parent	1-year follow-up

ABCD Sum Scores Culture & Environment Parent (abcd_sscep01)	nsc_p_ss_mean_3_items	Neighborhood Safety Protocol: mean of parent report: (neighborhood1r_p + neighborhood2r_p + neighborhood3r_p)/3	abcd_pnsc01	neighborhood1r_p	"I feel safe walking in my neighborhood, day or night"	Parent	1-year follow-up
	nsc_p_ss_mean_3_items	Neighborhood Safety Protocol: mean of parent report: (neighborhood1r_p + neighborhood2r_p + neighborhood3r_p)/3	abcd_pnsc01	neighborhood2r_p	"Violence is not a problem in my neighborhood"	Parent	1-year follow-up
				neighborhood3r_p	"My neighborhood is safe from crime"	Parent	1-year follow-up
ABCD Sum Scores Culture & Environment Parent (abcd_sscep01)	fes_p_ss_fc	Conflict subscale from the Family Environment Scale: sum of parent report (raw score): fam_enviro1_p + fam_enviro2r_p + fam_enviro3_p + fam_enviro4r_p + fam_enviro5_p + fam_enviro6_p + fam_enviro7r_p + fam_enviro8_p + fam_enviro9r_p	fes02	fam_enviro1_p	"We fight a lot in our family"	Parent	1-year follow-up
				fam_enviro2_p	"Family members rarely become openly angry"	Parent	1-year follow-up
				fam_enviro3_p	"Family members sometimes get so angry they throw things"	Parent	1-year follow-up
				fam_enviro4_p	"Family members hardly ever lose their tempers"	Parent	1-year follow-up
				fam_enviro5_p	"Family members often criticize each other"	Parent	1-year follow-up

				fam_enviro6_p	"Family members sometimes hit each other"	Parent	1-year follow-up
				fam_enviro7_p	"If there is a disagreement in our family, we try hard to smooth things over and keep the peace"	Parent	1-year follow-up
				fam_enviro8_p	"Family members often try to one-up or outdo each other"	Parent	1-year follow-up
				fam_enviro9_p	"In our family, we believe you don't ever get anywhere by raising your voice"	Parent	1-year follow-up
	fes_p_ss_fc_pr	Conflict subscale from the Family Environment Scale: sum of parent report (prorated score: Calculation: (fes_p_ss_fc)*(fes_p_ss_fc_nt)/(fes_p_ss_fc_na)	abcd_sscep01	fes_p_ss_fc	Conflict subscale from the Family Environment Scale: sum of parent report (raw score)	Parent	1-year follow-up
				fes_p_ss_fc_nt	Number of questions	Parent	1-year follow-up
				fes_p_ss_fc_na	Number of questions answered	Parent	1-year follow-up
	macv_p_ss_fs	MACVS Family Support subscale: mean: (mex_american2_p + mex_american7_p + mex_american12_p + mex_american16_p +	macv01	mex_american2_p	"Parents should teach their children that the family always comes first"	Parent	1-year follow-up
				mex_american7_p	"Family provides a sense of security because they will always be there for you"	Parent	1-year follow-up

	mex_american21_p + mex_american26_p)/6	mex_american12_p	"It is always important to be united as a family"	Parent	1-year follow-up
		mex_american16_p	"It is important to have close relationships with aunts/uncles, grandparents, and cousins"	Parent	1-year follow-up
		mex_american21_p	"Holidays and celebrations are important because the whole family comes together"	Parent	1-year follow-up
		mex_american26_p	"It is important for family members to show their love and affection to one another"	Parent	1-year follow-up
macv_p_ss_fo	MACVS Family Obligation subscale: mean: (mex_american3_p + mex_american8_p + mex_american13_p + mex_american17_p + mex_american22_p)/5	mex_american3_p	"Children should be taught that it is their duty to care for their parents when their parents get old"	Parent	1-year follow-up
		mex_american8_p	"If a relative is having a hard time financially, one should help them out if possible"	Parent	1-year follow-up
		mex_american13_p	"A person should share their home with relatives if they need a place to stay"	Parent	1-year follow-up
		mex_american17_p	"Older kids should take care of and be role models for their younger brothers and sisters"	Parent	1-year follow-up
		mex_american22_p	"Parents should be willing to make great sacrifices to make sure their children have a better life"	Parent	1-year follow-up

ABCD Sum Scores Culture & Environment Parent (abcd_sscep01)	macv_p_ss_isr	MACVS Independence & Self-Reliance subscale: mean: (mex_american5_p + mex_american10_p + mex_american14_p + mex_american19_p + mex_american24_p)/5	macv01	mex_american5_p	"People should learn how to take care of themselves and not depend on others"	Parent	1-year follow-up
				mex_american10_p	"The most important thing parents can teach their children is to be independent from others"	Parent	1-year follow-up
				mex_american14_p	"As children get older their parents should allow them to make their own decisions"	Parent	1-year follow-up
				mex_american19_p	"When there are problems in life, a person can only count on him or herself"	Parent	1-year follow-up
				mex_american24_p	"Parents should encourage children to solve their own problems"	Parent	1-year follow-up
	macv_p_ss_fr	MACVS Family as Referent subscale: mean: (mex_american4_p + mex_american9_p + mex_american18_p + mex_american23_p + mex_american27_p)/5		mex_american4_p	"Children should always do things to make their parents happy"	Parent	1-year follow-up
				mex_american9_p	"When it comes to important decisions, the family should ask for advice from close relatives"	Parent	1-year follow-up
				mex_american18_p	"Children should be taught to always be good because they represent the family"	Parent	1-year follow-up
				mex_american23_p	"A person should always think about their family when making important decisions"	Parent	1-year follow-up

			mex_american27_p	"It is important to work hard and do one's best because this work reflects on the family"	Parent	1-year follow-up
macv_p_ss_r	MACVS Religion subscale: mean: (mex_american1_p + mex_american6_p + mex_american11_p + mex_american15_p + mex_american20_p + mex_american25_p + mex_american28_p)/7		mex_american1_p	"One's belief in God gives inner strength and meaning to life"	Parent	1-year follow-up
			mex_american6_p	"God is first; family is second"	Parent	1-year follow-up
			mex_american11_p	"Parents should teach their children how to pray"	Parent	1-year follow-up
			mex_american15_p	"If everything is taken away, one still has their faith in God"	Parent	1-year follow-up
			mex_american20_p	"It is important to thank God every day for all one has"	Parent	1-year follow-up
			mex_american25_p	"It is important to follow the Word of God"	Parent	1-year follow-up
			mex_american28_p	"Religion should be an important part of one's life"	Parent	1-year follow-up
meim_p_ss_exp	MEIM-R Exploration subscale: mean: (meim_1_p + meim_4_p + meim_5_p)/3	abcd_meim01	meim_1_p	"I have spent time trying to find out more about my ethnic group, such as its history, traditions, and customs"	Parent	baseline

				meim_4_p	"I have often done things that will help me understand my ethnic background better"	Parent	baseline
				meim_5_p	"I have often talked to other people in order to learn more about my ethnic group"	Parent	baseline
	meim_p_ss_com	MEIM-R Commitment and Attachment subscale: mean: (meim_2_p + meim_3_p + meim_6_p)/3		meim_2_p	"I have a strong sense of belonging to my own ethnic group"	Parent	baseline
				meim_3_p	"I understand pretty well what my ethnic group membership means to me"	Parent	baseline
				meim_6_p	"I feel a strong attachment towards my own ethnic group"	Parent	baseline
	meim_p_ss_total	MEIM-R overall mean: (meim_1_p + meim_2_p + meim_3_p + meim_4_p + meim_5_p + meim_6_p)/6		meim_1_p	"I have spent time trying to find out more about my ethnic group, such as its history, traditions, and customs"	Parent	baseline
ABCD Sum Scores Culture & Environment Parent (abcd_sscep01)	meim_p_ss_total	MEIM-R overall mean: (meim_1_p + meim_2_p + meim_3_p + meim_4_p + meim_5_p + meim_6_p)/6	abcd_meim01	meim_2_p	"I have a strong sense of belonging to my own ethnic group"	Parent	baseline
				meim_3_p	"I understand pretty well what my ethnic group membership means to me"	Parent	baseline
				meim_4_p	"I have often done things that will help me understand my ethnic background better"	Parent	baseline

				meim_5_p	"I have often talked to other people in order to learn more about my ethnic group"	Parent	baseline
				meim_6_p	"I feel a strong attachment towards my own ethnic group"	Parent	baseline
ABCD Sum Scores Physical Health Parent (abcd_ssphp01)	cna_p_ss_sum	Mind Diet score: sum: cna_1_p + cna_2_p + cna_3_p + cna_4_p + cna_5_p + cna_6_p + cna_7_p + cna_8_p + cna_9_p + cna_10_p + cna_11_p + cna_12_p + cna_13_p + cna_14_p	abcd_cna01	cna_1_p	In a typical week, does your child eat whole grains 3 or more times per day?	Parent	1-year follow-up
				cna_2_p	In a typical week, does your child eat green leafy vegetables 6 or more times?	Parent	1-year follow-up
				cna_3_p	In a typical week, does your child eat other vegetables 1 or more time per day?	Parent	1-year follow-up
				cna_4_p	In a typical week, does your child eat berries 2 or more times?	Parent	1-year follow-up
				cna_5_p	In a typical week, does your child eat red meats and meat products less than 4 times?	Parent	1-year follow-up
				cna_6_p	In a typical week, does your child eat fish 1 or more times?	Parent	1-year follow-up
				cna_7_p	In a typical week, does your child eat poultry 2 or more times?	Parent	1-year follow-up
				cna_8_p	In a typical week, does your child eat beans 4 or more times?	Parent	1-year follow-up

				cna_9_p	In a typical week, does your child eat nuts 5 or more times?	Parent	1-year follow-up
				cna_10_p	In a typical week, does your child eat fast food or fried food less than 1 time?	Parent	1-year follow-up
				cna_11_p	In a typical week, does your child eat food in which olive oil is used as the primary oil?	Parent	1-year follow-up
				cna_12_p	In a typical week, does your child eat less than 1 tablespoon of butter or margarine per day?	Parent	1-year follow-up
				cna_13_p	In a typical week, does your child eat cheese less than 1 time?	Parent	1-year follow-up
				cna_14_p	In a typical week, does your child eat pastries or sweets less than 5 times?	Parent	1-year follow-up
ABCD Sum Scores Culture & Environment Youth (abcd_sscey01)	fes_y_ss_fc	Conflict subscale from the Family Environment Scale: sum of youth report (raw score): fes_youth_q1 + fes_youth_q2 + fes_youth_q3 + fes_youth_q4 + fes_youth_q5 + fes_youth_q6 + fes_youth_q7 + fes_youth_q8 + fes_youth_q9	abcd_fes01	fes_youth_q1	"We fight a lot in our family"	Youth	1-year follow-up
				fes_youth_q2	"Family members rarely become openly angry"	Youth	1-year follow-up
				fes_youth_q3	"Family members sometimes get so angry they throw things"	Youth	1-year follow-up

				fes_youth_q4	"Family members hardly ever lose their tempers"	Youth	1-year follow-up
				fes_youth_q5	"Family members often criticize each other"	Youth	1-year follow-up
ABCD Sum Scores Culture & Environment Youth (abcd_sscey01)	fes_y_ss_fc	Conflict subscale from the Family Environment Scale: sum of youth report (raw score): fes_youth_q1 + fes_youth_q2 + fes_youth_q3 + fes_youth_q4 + fes_youth_q5 + fes_youth_q6 + fes_youth_q7 + fes_youth_q8 + fes_youth_q9	abcd_fes01	fes_youth_q6	"Family members sometimes hit each other"	Youth	1-year follow-up
				fes_youth_q7	"If there's a disagreement in our family, we try hard to smooth things over and keep the peace"	Youth	1-year follow-up
				fes_youth_q8	"Family members often try to one-up or outdo each other"	Youth	1-year follow-up
				fes_youth_q9	"In our family, we believe you don't ever get anywhere by raising your voice"	Youth	1-year follow-up
	fes_y_ss_fc_pr	Conflict subscale from the Family Environment Scale: sum of youth report (prorated score): (fes_y_ss_fc)*(fes_y_ss_fc_nt)/(fes_y_ss_fc_na)	abcd_sscey01	fes_y_ss_fc	Conflict subscale from the Family Environment Scale: sum of youth report (raw score)	Youth	1-year follow-up
				fes_y_ss_fc_nt	Number of questions	Youth	1-year follow-up
				fes_y_ss_fc_na	Number of questions answered	Youth	1-year follow-up

	crpbi_y_ss_parent	CRPBI Acceptance subscale: mean of primary caregiver report by youth: (crpbi_parent1_y + crpbi_parent2_y + crpbi_parent3_y, crpbi_parent4_y + crpbi_parent5_y)/5	crpbi01	crpbi_parent1_y	"First caregiver makes me feel better after talking over my worries with him/her"	Youth	1-year follow-up
				crpbi_parent2_y	"First caregiver smiles at me very often"	Youth	1-year follow-up
				crpbi_parent3_y	"First caregiver is able to make me feel better when I am upset"	Youth	1-year follow-up
				crpbi_parent4_y	"First caregiver believes in showing his/her love for me"	Youth	1-year follow-up
				crpbi_parent5_y	"First caregiver is easy to talk to"	Youth	1-year follow-up
	crpbi_y_ss_caregiver	CRPBI Acceptance subscale: mean of secondary caregiver report by youth: (crpbi_caregiver12_y + crpbi_caregiver13_y + crpbi_caregiver14_y + crpbi_caregiver15_y + crpbi_caregiver16_y)/5		crpbi_parent12_y	"Second caregiver makes me feel better after talking over my worries with him/her"	Youth	1-year follow-up
				crpbi_parent13_y	"Second caregiver smiles at me very often"	Youth	1-year follow-up
				crpbi_parent14_y	"Second caregiver is able to make me feel better when I am upset"	Youth	1-year follow-up
				crpbi_parent15_y	"Second caregiver believes in showing his/her love for me"	Youth	1-year follow-up

				crpbi_parent16_y	"Second caregiver is easy to talk to"	Youth	1-year follow-up
	pmq_y_ss_mean	Parental Monitoring score: mean: (parent_monitor_q1_y + parent_monitor_q2_y + parent_monitor_q3_y + parent_monitor_q4_y parent_monitor_q5_y)/5	pmq01	parent_monitor_q1_y	How often do your parents/guardians know where you are?	Youth	1-year follow-up
				parent_monitor_q2_y	How often do your parents know who you are with when you are not at school and away from home?	Youth	1-year follow-up
				parent_monitor_q3_y	If you are at home when your parents or guardians are not, how often do you know how to get in touch with them?	Youth	1-year follow-up
				parent_monitor_q4_y	How often do you talk to your mom/dad or guardian about your plans for the coming day, such as your plans about what will happen at school or what you are going to do with friends?	Youth	1-year follow-up
				parent_monitor_q5_y	In an average week, how many times do you and your parents/guardians, eat dinner together?	Youth	1-year follow-up
ABCD Parent Adult Self Report Scores Aseba (ASR) (abcd_asrs01)	asr_scr_internal_t	Internalizing Problems ASR Syndrome Scale (t-score)	pasr01	asr_q14_p	I cry a lot	Parent	baseline
	asr_scr_depress_t	Depressive Problems ASR DSM-5-Oriented Scale (t-score)		asr_q18_p	I deliberately try to hurt or kill myself	Parent	baseline
				asr_q24_p	I don't eat as well as I should	Parent	baseline

ABCD Parent Adult Self Report Scores Aseba (ASR) (abcd_asrs01)	asr_scr_depress_t	Depressive Problems ASR DSM-5-Oriented Scale (t-score)	pasr01	asr_q35_p	I feel worthless and inferior	Parent	baseline
				asr_q52_p	I feel too guilty	Parent	baseline
				asr_q54_p	I feel tired without good reason	Parent	baseline
				asr_q60_p	There is very little I enjoy	Parent	baseline
				asr_q77_p	I sleep more than most other people during day and/or night	Parent	baseline
				asr_q78_p	I have trouble making decisions	Parent	baseline
				asr_q91_p	I think about killing myself	Parent	baseline
				asr_q96_p	I think about sex too much	Parent	baseline
				asr_q100_p	I have trouble sleeping	Parent	baseline
				asr_q102_p	I don't have much energy	Parent	baseline
				asr_q103_p	I am unhappy, sad, or depressed	Parent	baseline
				asr_q107_p	I feel that I can't succeed	Parent	baseline
	asr_scr_anxdisord_t	Anxiety Problems ASR DSM-5-Oriented Scale (t-score)		asr_q22_p	I worry about my future	Parent	baseline
				asr_q29_p	I am afraid of certain animals, situations, or places	Parent	baseline
				asr_q45_p	I am nervous or tense	Parent	baseline
				asr_q50_p	I am too fearful or anxious	Parent	baseline
				asr_q56h_p	Heart pounding or racing	Parent	baseline
				asr_q72_p	I worry about my family	Parent	baseline
				asr_q112_p	I worry a lot	Parent	baseline
				asr_q51_p	I feel dizzy or lightheaded	Parent	baseline

asr_scr_somaticpr_t	Somatic Problems ASR DSM-5-Oriented Scale (t-score)	asr_q56a_p	Aches or pains (not stomach or headaches)	Parent	baseline
		asr_q56b_p	Headaches	Parent	baseline
		asr_q56c_p	Nausea, feel sick	Parent	baseline
		asr_q56d_p	Problems with eyes (not if corrected by glasses)	Parent	baseline
		asr_q56e_p	Rashes or other skin problems	Parent	baseline
		asr_q56f_p	Stomachaches	Parent	baseline
		asr_q56g_p	Vomiting, throwing up	Parent	baseline
		asr_q56i_p	Numbness or tingling in body parts	Parent	baseline
asr_scr_external_t	Externalizing Problems ASR Syndrome Scale (t-score)	asr_q25_p	I don't get along with other people	Parent	baseline
asr_scr_avoidant_t	Avoidant Personality Problems ASR DSM-5-Oriented Scale (t-score)	asr_q42_p	I would rather be alone than with others	Parent	baseline
		asr_q47_p	I lack self-confidence	Parent	baseline
		asr_q67_p	I have trouble making or keeping friends	Parent	baseline
		asr_q71_p	I am self-conscious or easily embarrassed	Parent	baseline
		asr_q75_p	I am too shy or timid	Parent	baseline
		asr_q111_p	I keep from getting involved with others	Parent	baseline
		asr_q03_p	I argue a lot	Parent	baseline

	asr_scr_antisocial_t	Antisocial Personality Problems ASR DSM-5-Oriented Scale (t-score)		asr_q05_p	I blame others for my problems	Parent	baseline
				asr_q16_p	I am mean to others	Parent	baseline
ABCD Parent Adult Self Report Scores Aseba (ASR) (abcd_asrs01)	asr_scr_antisocial_t	Antisocial Personality Problems ASR DSM-5-Oriented Scale (t-score)	pasr01	asr_q21_p	I damage or destroy things belonging to others	Parent	baseline
				asr_q23_p	I break rules at work or elsewhere	Parent	baseline
				asr_q26_p	I don't feel guilty after doing something I shouldn't	Parent	baseline
				asr_q28_p	I get along badly with my family	Parent	baseline
				asr_q37_p	I get in many fights	Parent	baseline
				asr_q39_p	I hang around people who get into trouble	Parent	baseline
				asr_q43_p	I lie or cheat	Parent	baseline
				asr_q57_p	I physically attack people	Parent	baseline
				asr_q76_p	My behavior is irresponsible	Parent	baseline
				asr_q82_p	I steal	Parent	baseline
				asr_q92_p	I do things that may cause me trouble with the law	Parent	baseline
				asr_q95_p	I have a hot temper	Parent	baseline
				asr_q97_p	I threaten to hurt people	Parent	baseline
				asr_q101_p	I stay away from my job even when I'm not sick or not on vacation	Parent	baseline
				asr_q114_p	I fail to pay my debts or meet other financial responsibilities	Parent	baseline

		asr_q120_p	I drive too fast	Parent	baseline
		asr_q122_p	I have trouble keeping a job	Parent	baseline
asr_scr_adhd_t	ADHD Problems ASR DSM-5-Oriented Scale (t-score)	asr_q01_p	I am too forgetful	Parent	baseline
asr_scr_inattention_t	Inattention ASR DSM-5-Oriented Scale (t-score)	asr_q08_p	I have trouble concentrating or paying attention for long	Parent	baseline
		asr_q59_p	I fail to finish things I should do	Parent	baseline
		asr_q61_p	My work performance is poor	Parent	baseline
		asr_q108_p	I tend to lose things	Parent	baseline
		asr_q119_p	I am not good at details	Parent	baseline
asr_scr_hyperactive_t	Hyperactivity-Impulsivity ASR DSM-5-Oriented Scale (t-score)	asr_q10_p	I have trouble sitting still	Parent	baseline
		asr_q36_p	I accidentally get hurt a lot, accident-prone	Parent	baseline
		asr_q41_p	I am impulsive or act without thinking	Parent	baseline
		asr_q89_p	I rush into things without considering the risks	Parent	baseline
		asr_q105_p	People think I am disorganized	Parent	baseline
		asr_q115_p	I feel restless or fidgety	Parent	baseline
		asr_q118_p	I am too impatient	Parent	baseline
asr_scr_totprob_t	Total Problems ASR Syndrome Scale (t-score)	asr_q02_p	I make good use of my opportunities	Parent	baseline
		asr_q04_p	I work up to my ability	Parent	baseline

				asr_q06_p	I use drugs (other than alcohol, nicotine) for nonmedical purposes	Parent	baseline
				asr_q07_p	I brag	Parent	baseline
				asr_q09_p	I can't get my mind off certain thoughts	Parent	baseline
				asr_q11_p	I am too dependent on others	Parent	baseline
				asr_q12_p	I feel lonely	Parent	baseline
ABCD Parent Adult Self Report Scores Aseba (ASR) (abcd_asrs01)	asr_scr_totprob_t	Total Problems ASR Syndrome Scale (t-score)	pasr01	asr_q13_p	I feel confused or in a fog	Parent	baseline
				asr_q15_p	I am pretty honest	Parent	baseline
				asr_q17_p	I daydream a lot	Parent	baseline
				asr_q19_p	I try to get a lot of attention	Parent	baseline
				asr_q20_p	I damage or destroy my things	Parent	baseline
				asr_q27_p	I am jealous of others	Parent	baseline
				asr_q30_p	My relations with the opposite sex are poor	Parent	baseline
				asr_q31_p	I am afraid I might think or do something bad	Parent	baseline
				asr_q32_p	I feel that I have to be perfect	Parent	baseline
				asr_q33_p	I feel that no one loves me	Parent	baseline
				asr_q34_p	I feel that others are out to get me	Parent	baseline
				asr_q38_p	My relations with neighbors are poor	Parent	baseline

	asr_q39_p	I hang around people who get into trouble	Parent	baseline
	asr_q40_p	I hear sounds and voices that other people think aren't there	Parent	baseline
	asr_q44_p	I feel overwhelmed by my responsibilities	Parent	baseline
	asr_q46_p	Parts of my body twitch or make nervous movements	Parent	baseline
	asr_q48_p	I am not liked by others	Parent	baseline
	asr_q49_p	I can do certain things better than other people	Parent	baseline
	asr_q53_p	I have trouble planning for the future	Parent	baseline
	asr_q55_p	My moods swing between elation and depression	Parent	baseline
	asr_q58_p	I pick my skin or other parts of my body	Parent	baseline
	asr_q62_p	I am poorly coordinated or clumsy	Parent	baseline
	asr_q63_p	I would rather be with older people than with people of my own age	Parent	baseline
	asr_q64_p	I have trouble setting priorities	Parent	baseline
	asr_q65_p	I refuse to talk	Parent	baseline
	asr_q66_p	I repeat certain acts over and over	Parent	baseline
	asr_q68_p	I scream or yell a lot	Parent	baseline

				asr_q69_p	I am secretive or keep things to myself	Parent	baseline
				asr_q70_p	I see things that other people think aren't there	Parent	baseline
				asr_q73_p	I meet my responsibilities to my family	Parent	baseline
				asr_q74_p	I show off or clown	Parent	baseline
				asr_q79_p	I have a speech problem	Parent	baseline
				asr_q80_p	I stand up for my rights	Parent	baseline
				asr_q81_p	My behavior is very changeable	Parent	baseline
				asr_q83_p	I am easily bored	Parent	baseline
				asr_q84_p	I do things that other people think are strange	Parent	baseline
				asr_q85_p	I have thoughts that other people would think are strange	Parent	baseline
				asr_q86_p	I am stubborn, sullen, or irritable	Parent	baseline
ABCD Parent Adult Self Report Scores Aseba (ASR) (abcd_asrs01)	asr_scr_totprob_t	Total Problems ASR Syndrome Scale (t-score)	pasr01	asr_q87_p	My moods or feeling change suddenly	Parent	baseline
				asr_q88_p	I enjoy being with people	Parent	baseline
				asr_q90_p	I drink too much alcohol or get drunk	Parent	baseline
				asr_q93_p	I talk too much	Parent	baseline
				asr_q94_p	I tease others a lot	Parent	baseline
				asr_q98_p	I like to help others	Parent	baseline

asr_q99_p	I dislike staying in one place for very long	Parent	baseline
asr_q104_p	I am louder than others	Parent	baseline
asr_q106_p	I try to be fair to others	Parent	baseline
asr_q109_p	I like to try new things	Parent	baseline
asr_q110_p	I wish I were of the opposite sex	Parent	baseline
asr_q113_p	I worry about my relations with the opposite sex	Parent	baseline
asr_q116_p	I get upset too easily	Parent	baseline
asr_q117_p	I have trouble managing my money or credit card	Parent	baseline
asr_q121_p	I tend to be late for appointments	Parent	baseline
asr_q123_p	I am a happy person	Parent	baseline
asr_q124_p	In the past 6 months, about how many times per day did you use tobacco (including smokeless tobacco)?	Parent	baseline
asr_q125_p	In the past 6 months, on how many days were you drunk?	Parent	baseline
asr_q126_p	In the past 6 months, on how many days did you use drugs for nonmedical purposes (including marijuana, cocaine, and other drugs, except alcohol and nicotine)?	Parent	baseline

Sum Scores Traumatic Brain Injury (abcd_tbi01)	tbi_ss_nmrpi	Number of periods with multiple or repeated injuries: tbi_7a + tbi_7g + tbi_8g	abcd_otbi01	tbi_7a	Did your child experience a period of time in his/her life when he/she experienced multiple, repeated impacts to the head? (e.g., abused, contact sports)	Parent	baseline
				tbi_7g	Was there another instance where your child experienced a period of time in his/her life when he/she experienced multiple, repeated impacts to the head?	Parent	baseline
				tbi_8g	Was there another instance where your child experienced a period of time in his/her life when he/she experienced multiple, repeated impacts to the head?	Parent	baseline
	tbi_ss_worst_overall	Worst Injury Overall (Improbable TBI; Possible mild TBI; Mild TBI; Moderate TBI; Severe TBI)		tbi_1	Has your child ever been hospitalized or treated in an emergency room following an injury to his/her head or neck?	Parent	baseline
				tbi_1b	Was he/she knocked out or did he/she lose consciousness (LOC)? If yes, how long?	Parent	baseline
				tbi_1c	Was he/she dazed or did he/she have a gap in his/her memory from the injury?	Parent	baseline
				tbi_2	Has your child ever injured his/her head or neck in a car accident or from crashing some other moving vehicle like a bicycle, motorcyle, or ATV?	Parent	baseline

				tbi_2b	Was he/she knocked out or did he/she lose consciousness (LOC)? If yes, how long?	Parent	baseline
Sum Scores Traumatic Brain Injury (abcd_tbi01)	tbi_ss_worst_overall	Worst Injury Overall (Improbable TBI; Possible mild TBI; Mild TBI; Moderate TBI; Severe TBI)	abcd_otbi01	tbi_2c	Was he/she dazed or did he/she have a gap in his/her memory from the injury?	Parent	baseline
				tbi_3	Has you child ever injured his/her head or neck in a fall or from being hit by something? (For example, falling from a bike or horse, rollerblading, falling on ice, being hit by a rock) Has your child ever injured his/her head or neck playing sports or on the playground?	Parent	baseline
				tbi_3b	Was he/she knocked out or did he/she lose consciousness (LOC)? If yes, how long?	Parent	baseline
				tbi_3c	Was he/she dazed or did he/she have a gap in his/her memory from the injury?	Parent	baseline
				tbi_4	Has your child ever injured his/her head or neck in a fight, from being hit by something, or from being shaken violently? Has your child ever been shot in the head?	Parent	baseline
				tbi_4b	Was he/she knocked out or did he/she lose consciousness (LOC)? If yes, how long?	Parent	baseline

	tbi_4c	Was he/she dazed or did he/she have a gap in his/her memory from the injury?	Parent	baseline
	tbi_5	Has your child ever been nearby when an explosion or blast has occurred?	Parent	baseline
	tbi_5b	Was he/she knocked out or did he/she lose consciousness (LOC)? If yes, how long?	Parent	baseline
	tbi_5c	Was he/she dazed or did he/she have a gap in his/her memory from the injury?	Parent	baseline
	tbi_6o	Do you want to report any more injuries with LOC?	Parent	baseline
	tbi_6p	If yes, how many more?	Parent	baseline
	tbi_6q	How many minutes was the longest knock out?	Parent	baseline
	tbi_6r	How many were more than or equal to 30 min?	Parent	baseline
	tbi_7a	Did your child experience a period of time in his/her life when he/she experienced multiple, repeated impacts to the head? (e.g., abused, contact sports)	Parent	baseline
	tbi_7c1	Was he/she knocked out or did he/she lose consciousness (LOC)? If yes, how long?	Parent	baseline

				tbl_7c2	Was he/she dazed or did he/she have a gap in his/her memory from the injury?	Parent	baseline
				tbi_7e	At what age did these effects begin?	Parent	baseline
				tbi_7f	At what age did these effects end?	Parent	baseline
				tbi_7g	Was there another instance where your child experienced a period of time in his/her life when he/she experienced multiple, repeated impacts to the head?	Parent	baseline
				tbi_7i	What was the typical effect of the injury?	Parent	baseline
				tbi_7k	At what age did these effects begin?	Parent	baseline
				tbi_7l	At what age did these effects end?	Parent	baseline
Sum Scores Traumatic Brain Injury (abcd_tbi01)	tbi_ss_worst_overall	Worst Injury Overall (Improbable TBI; Possible mild TBI; Mild TBI; Moderate TBI; Severe TBI)	abcd_otbi01	tbi_8g	Was there another instance where your child experienced a period of time in his/her life when he/she experienced multiple, repeated impacts to the head?	Parent	baseline
				tbi_8i	What was the typical effect of the injury?	Parent	baseline
				tbi_8k	At what age did these effects begin?	Parent	baseline

			tbi_8l	At what age did these effects end?	Parent	baseline
ABCD Parent Community Risk and Protective Factors (CRPF) (abcd_crpf01)	su_risk_p_1	If your child wanted to get some beer, wine, or hard liquor (for example vodka, whiskey, or gin), how easy would it be for her/him to get some?			Parent	1-year follow-up
	su_risk_p_2	If your child wanted to get some cigarettes, how easy would it be for her/him to get some?			Parent	1-year follow-up
	su_risk_p_3	If your child wanted to get some e-cigarettes, vape pens, or e-hookah, how easy would it be for her/him to get some?			Parent	1-year follow-up
	su_risk_p_4	If your child wanted to get some marijuana, how easy would it be for her/him to get some?			Parent	1-year follow-up
	su_risk_p_5	If your child wanted to get a drug like cocaine, LSD, or amphetamines, how easy would it be for her/him to get some?			Parent	1-year follow-up
	su_risk_p_6	Is "medical marijuana" (marijuana prescribed by a doctor) legal in your state?			Parent	1-year follow-up
ABCD Youth Neighborhood Safety/Crime Survey Modified from PhenX	neighborhood_crime_y	"My neighborhood is safe from crime"			Youth	1-year follow-up

(NSC) (abcd_nsc01)				
ABCD Youth Screen Time Survey (STQ) (abcd_stq01)	screen1_wkdy_y	On a typical weekday, how many hours do you watch TV shows or movies?	Youth	1-year follow-up
	screen2_wkdy_y	On a typical weekday, how many hours do you watch videos (such as YouTube)?	Youth	1-year follow-up
	screen3_wkdy_y	On a typical weekday, how many hours do you play video games on a computer, console, phone or other device (Xbox, Play Station, iPad)?	Youth	1-year follow-up
	screen4_wkdy_y	On a typical weekday, how many hours do you text on a cell phone, tablet, or computer (e.g. GChat, Whatsapp, etc.)?	Youth	1-year follow-up
	screen5_wkdy_y	On a typical weekday, how many hours do you visit social networking sites like Facebook, Twitter, Instagram, etc.?	Youth	1-year follow-up
	screen_wkdy_y	On a typical weekday, how many hours do you video chat (Skype, Facetime, etc.)?	Youth	1-year follow-up
	screen7_wknd_y	On a typical weekend day, how many hours do you watch TV shows or movies?	Youth	1-year follow-up
	screen8_wknd_y	On a typical weekend day, how many hours do you watch videos (such as YouTube)?	Youth	1-year follow-up

	screen9_wknd_y	On a typical weekend day, how many hours do you play video games on a computer, console, phone or other device (Xbox, Play Station, iPad)?	Youth	1-year follow-up
ABCD Youth Screen Time Survey (STQ) (abcd_stq01)	screen10_wknd_y	On a typical weekend day, how many hours do you text on a cell phone, tablet, or computer (GChat, Whatsapp, etc.)?	Youth	1-year follow-up
	screen11_wknd_y	On a typical weekend day, how many hours do you visit social networking sites like Facebook, Twitter, Instagram, etc.?	Youth	1-year follow-up
	screen12_wknd_y	On a typical weekend day, how many hours do you video chat (Skype, Facetime, etc.)?	Youth	1-year follow-up
	screen13_y	How often do you play mature-rated video games (e.g., Call of Duty, Grand Theft Auto, Assassin's Creed, etc.)?	Youth	1-year follow-up
	screen14_y	How often do you watch R-rated movies?	Youth	1-year follow-up
ABCD Parental Monitoring Survey (pmq01)	parent_monitor_q1_y	How often do your parents/guardians know where you are?	Youth	1-year follow-up
	parent_monitor_q2_y	How often do your parents know who you are with when you are not at school and away from home?	Youth	1-year follow-up
	parent_monitor_q3_y	If you are at home when your parents or guardians are not, how often do you know how to get in touch with them?	Youth	1-year follow-up

	parent_monitor_q4_y	How often do you talk to your mom/dad or guardian about your plans for the coming day, such as your plans about what will happen at school or what you are going to do with friends?	Youth	1-year follow-up
	parent_monitor_q5_y	In an average week, how many times do you and your parents/guardians, eat dinner together?	Youth	1-year follow-up
ABCD Parental Rules on Substance Use (prq01)	parent_rules_q1	What are the family rules about drinking alcohol for your son/daughter?	Parent	1-year follow-up
	parent_rules_q1a	Are these the same rules for all family members?	Parent	1-year follow-up
	parent_rules_q2	Do you have penalties for violating family rules about drinking?	Parent	1-year follow-up
	parent_rules_q4	What are the family rules about smoking cigarettes for your son/daughter?	Parent	1-year follow-up
	parent_rules_q5	Are these the same rules for all family members?	Parent	1-year follow-up
	parent_rules_q6	Do you enforce penalties for violating family rules about smoking?	Parent	1-year follow-up
	parent_rules_q7	What are the family rules about using marijuana for your son/daughter?	Parent	1-year follow-up

ABCD Youth Discrimination Measure (abcd_ydmes01)	dim_yesno_q1	In the past 12 months, have you felt discriminated against because of your race, ethnicity, or color?	Youth	1-year follow-up
	dim_yesno_q2	In the past 12 months, have you felt discriminated against because you are (or your family is) from another country?	Youth	1-year follow-up
	dim_yesno_q3	In the past 12 months, have you felt discriminated against because someone thought you were gay, lesbian, or bisexual?	Youth	1-year follow-up
	dim_yesno_q4	In the past 12 months, have you felt discriminated against because of your weight?	Youth	1-year follow-up
	dim_matrix_q1	How often do teachers treat you unfairly or negatively because of your ethnic background?	Youth	1-year follow-up
ABCD Youth Discrimination Measure (abcd_ydmes01)	dim_matrix_q2	How often do other adults outside school treat you unfairly or negatively because of your ethnic background?	Youth	1-year follow-up
	dim_matrix_q3	How often do other students treat you unfairly or negatively because of your ethnic background?	Youth	1-year follow-up
	dim_matrix_q4	"I feel that others behave in an unfair or negative way toward my ethnic group"	Youth	1-year follow-up
	dim_matrix_q5	"I feel that I am not wanted in American society"	Youth	1-year follow-up

	dim_matrix_q6	"I don't feel accepted by other Americans"	Youth	1-year follow-up
	dim_matrix_q7	"I feel that other Americans have something against me"	Youth	1-year follow-up
ABCD School Risk and Protective Factors Survey (srpf01)	school_2_y	"In my school, students have lots of chances to help decide things like class activities and rules"	Youth	1-year follow-up
	school_3_y	"I get along with my teachers"	Youth	1-year follow-up
	school_4_y	"My teacher(s) notices when I am doing a good job and lets me know about it"	Youth	1-year follow-up
	school_5_y	"There are lots of chances for students in my school to get involved in sports, clubs, or other school activities outside of class"	Youth	1-year follow-up
	school_6_y	"I feel safe at my school"	Youth	1-year follow-up
	school_7_y	"The school lets my parents know when I have done something well"	Youth	1-year follow-up
	school_8_y	"I like school because I do well in class"	Youth	1-year follow-up

	school_9_y	"I feel I'm just as smart as other kids my age"	Youth	1-year follow-up
	school_10_y	"There are lots of chances to be part of class discussions or activities"	Youth	1-year follow-up
	school_12_y	"In general, I like school a lot"	Youth	1-year follow-up
	school_15_y	"Usually, school bores me"	Youth	1-year follow-up
	school_17_y	"Getting good grades is not so important to me"	Youth	1-year follow-up
ABCD Parent Acculturation Survey Modified from PhenX (ACC) (pacc01)	accult_q1_p	How well do you speak English?	Parent	1-year follow-up
	accult_q2_p	Besides English, do you speak or understand another language or dialect?	Parent	1-year follow-up
ABCD Youth Acculturation Survey Modified from PhenX (ACC) (yacc01)	accult_q1_y	How well do you speak English?	Youth	1-year follow-up
	accult_q2_y	Besides English, do you speak or understand another language or dialect?	Youth	1-year follow-up

ABCD Parent Family History Summary Scores (abcd_fhxssp01)	famhx_ss_fath_prob_alc_p	Father alcohol problem	fhxp102	famhx_4_p	Has ANY blood relative of your child ever had any problems due to alcohol, such as: Marital separation or divorce; Laid off or fired from work; Arrests or DUIs; Alcohol harmed their health; In an alcohol treatment program; Suspended or expelled from school 2 or more times; Isolated self from family, caused arguments or were drunk a lot.	Parent	baseline
				famhx4a_p___1	Biological father (Marital)	Parent	baseline
				famhx4a_p___2	Biological father (Work)	Parent	baseline
				famhx4a_p___3	Biological father (Arrests/DUI)	Parent	baseline
				famhx4a_p___7	Biological father (Health)	Parent	baseline
				famhx4a_p___4	Biological father (Alcohol treatment program)	Parent	baseline
				famhx4a_p___5	Biological father (School)	Parent	baseline
				famhx4a_p___6	Biological father (Isolated self, arguments, drunk a lot)	Parent	baseline
	famhx_ss_patgf_prob_alc_p	Paternal grandfather alcohol problem		famhx4b_p___1	Paternal grandfather (Marital)	Parent	baseline
				famhx4b_p___2	Paternal grandfather (Work)	Parent	baseline
				famhx4b_p___3	Paternal grandfather (Arrests/DUI)	Parent	baseline
				famhx4b_p___7	Paternal grandfather (Health)	Parent	baseline
				famhx4b_p___4	Paternal grandfather (Alcohol treatment program)	Parent	baseline

		famhx4b_p___5	Paternal grandfather (School)	Parent	baseline
		famhx4b_p___6	Paternal grandfather (Isolated self, arguments, drunk a lot)	Parent	baseline
famhx_ss_patgm_prob_alc_p	Paternal grandmother alcohol problem	famhx_4c_p___1	Paternal grandmother (Marital)	Parent	baseline
		famhx_4c_p___2	Paternal grandmother (Work)	Parent	baseline
		famhx_4c_p___3	Paternal grandmother (Arrests/DUI)	Parent	baseline
		famhx_4c_p___7	Paternal grandmother (Health)	Parent	baseline
		famhx_4c_p___4	Paternal grandmother (Alcohol treatment program)	Parent	baseline
		famhx_4c_p___5	Paternal grandmother (School)	Parent	baseline
		famhx_4c_p___6	Paternal grandmother (Isolated self, arguments, drunk a lot)	Parent	baseline
famhx_ss_moth_prob_alc_p	Mother alcohol problem	famhx_4d_p___1	Biological mother (Marital)	Parent	baseline
		famhx_4d_p___2	Biological mother (Work)	Parent	baseline
		famhx_4d_p___3	Biological mother (Arrests/DUI)	Parent	baseline
		famhx_4d_p___7	Biological mother (Health)	Parent	baseline
		famhx_4d_p___4	Biological mother (Alcohol treatment program)	Parent	baseline
		famhx_4d_p___5	Biological mother (School)	Parent	baseline
		famhx_4d_p___6	Biological mother (Isolated self, arguments, drunk a lot)	Parent	baseline
	Maternal grandfather alcohol problem	famhx_4e_p___1	Maternal grandfather (Marital)	Parent	baseline

	famhx_ss_matgf_prob_alc_p			famhx_4e_p___2	Maternal grandfather (Work)	Parent	baseline
				famhx_4e_p___3	Maternal grandfather (Arrests/DUI)	Parent	baseline
				famhx_4e_p___7	Maternal grandfather (Health)	Parent	baseline
				famhx_4e_p___4	Maternal grandfather (Alcohol treatment program)	Parent	baseline
ABCD Parent Family History Summary Scores (abcd_fhxssp01)	famhx_ss_matgf_prob_alc_p	Maternal grandfather alcohol problem	fhxp102	famhx_4e_p___5	Maternal grandfather (School)	Parent	baseline
				famhx_4e_p___6	Maternal grandfather (Isolated self, arguments, drunk a lot)	Parent	baseline
	famhx_ss_matgm_prob_alc_p	Maternal grandmother alcohol problem		famhx_4f_p___1	Maternal grandmother (Marital)	Parent	baseline
				famhx_4f_p___2	Maternal grandmother (Work)	Parent	baseline
				famhx_4f_p___3	Maternal grandmother (Arrests/DUI)	Parent	baseline
				famhx_4f_p___7	Maternal grandmother (Health)	Parent	baseline
				famhx_4f_p___4	Maternal grandmother (Alcohol treatment program)	Parent	baseline
				famhx_4f_p___5	Maternal grandmother (School)	Parent	baseline
				famhx_4f_p___6	Maternal grandmother (Isolated self, arguments, drunk a lot)	Parent	baseline
	famhx_ss_fath_prob_dg_p	Father drug use problem		fam_history_5_yes_no	Has ANY blood relative of your child ever had any problems due to drugs, such as: Marital separation or divorce; Laid off or fired from work; Arrests or DUIs; Drugs harmed their health; In a drug treatment program; Suspended or expelled from	Parent	baseline

			school 2 or more times; Isolated self from family, caused arguments or were high a lot.		
		fam_history_q5a _drugs___1	Biological father (Marital)	Parent	baseline
		fam_history_q5a _drugs___2	Biological father (Work)	Parent	baseline
		fam_history_q5a _drugs___3	Biological father (Arrests/DUI)	Parent	baseline
		fam_history_q5a _drugs___7	Biological father (Health)	Parent	baseline
		fam_history_q5a _drugs___4	Biological father (Drug treatment program)	Parent	baseline
		fam_history_q5a _drugs___5	Biological father (School)	Parent	baseline
		fam_history_q5a _drugs___6	Biological father (Isolated self, arguments, high a lot)	Parent	baseline
famhx_ss_patgf_prob_dg_p	Paternal grandfather drug use problem	fam_history_q5b _drugs___1	Paternal grandfather (Marital)	Parent	baseline
		fam_history_q5b _drugs___2	Paternal grandfather (Work)	Parent	baseline
		fam_history_q5b _drugs___3	Paternal grandfather (Arrests/DUI)	Parent	baseline

				fam_history_q5b_drugs___7	Paternal grandfather (Health)	Parent	baseline
				fam_history_q5b_drugs___4	Paternal grandfather (Drug treatment program)	Parent	baseline
				fam_history_q5b_drugs___5	Paternal grandfather (School)	Parent	baseline
				fam_history_q5b_drugs___6	Paternal grandfather (Isolated self, arguments, high a lot)	Parent	baseline
	famhx_ss_patgm_prob_dg_p	Paternal grandmother drug use problem		fam_history_q5c_drugs___1	Paternal grandmother (Marital)	Parent	baseline
				fam_history_q5c_drugs___2	Paternal grandmother (Work)	Parent	baseline
				fam_history_q5c_drugs___3	Paternal grandmother (Arrests/DUI)	Parent	baseline
				fam_history_q5c_drugs___7	Paternal grandmother (Health)	Parent	baseline
				fam_history_q5c_drugs___4	Paternal grandmother (Drug treatment program)	Parent	baseline
				fam_history_q5c_drugs___5	Paternal grandmother (School)	Parent	baseline
				fam_history_q5c_drugs___6	Paternal grandmother (Isolated self, arguments, high a lot)	Parent	baseline
ABCD Parent Family History Summary Scores	famhx_ss_moth_prob_dg_p	Mother drug use problem	fhxp102	fam_history_q5d_drugs___1	Biological mother (Marital)	Parent	baseline
				fam_history_q5d_drugs___2	Biological mother (Work)	Parent	baseline

(abcd_fhxssp01)			fam_history_q5d_drugs___3	Biological mother (Arrests/DUI)	Parent	baseline
			fam_history_q5d_drugs___7	Biological mother (Health)	Parent	baseline
			fam_history_q5d_drugs___4	Biological mother (Drug treatment program)	Parent	baseline
			fam_history_q5d_drugs___5	Biological mother (School)	Parent	baseline
			fam_history_q5d_drugs___6	Biological mother (Isolated self, arguments, high a lot)	Parent	baseline
	famhx_ss_matgf_prob_dg_p	Maternal grandfather drug use problem	fam_history_q5e_drugs___1	Maternal grandfather (Marital)	Parent	baseline
			fam_history_q5e_drugs___2	Maternal grandfather (Work)	Parent	baseline
			fam_history_q5e_drugs___3	Maternal grandfather (Arrests/DUI)	Parent	baseline
			fam_history_q5e_drugs___7	Maternal grandfather (Health)	Parent	baseline
			fam_history_q5e_drugs___4	Maternal grandfather (Drug treatment program)	Parent	baseline
			fam_history_q5e_drugs___5	Maternal grandfather (School)	Parent	baseline
			fam_history_q5e_drugs___6	Maternal grandfather (Isolated self, arguments, high a lot)	Parent	baseline
	famhx_ss_matgm_prob_dg_p	Maternal grandmother drug use problem	fam_history_q5f_drugs___1	Maternal grandmother (Marital)	Parent	baseline

			fam_history_q5f_drugs___2	Maternal grandmother (Work)	Parent	baseline
			fam_history_q5f_drugs___3	Maternal grandmother (Arrests/DUI)	Parent	baseline
			fam_history_q5f_drugs___7	Maternal grandmother (Health)	Parent	baseline
			fam_history_q5f_drugs___4	Maternal grandmother (Drug treatment program)	Parent	baseline
			fam_history_q5f_drugs___5	Maternal grandmother (School)	Parent	baseline
			fam_history_q5f_drugs___6	Maternal grandmother (Isolated self, arguments, high a lot)	Parent	baseline
	famhx_ss_fath_prob_dprs_p	Father depression problem	fam_history_6_yes_no	Has ANY blood relative of your child ever suffered from depression, that is, have they felt so low for a period of at least two weeks that they hardly ate or slept or couldn't work or do whatever they usually do?	Parent	baseline
			fam_history_q6a_depression	Biological father	Parent	baseline
	famhx_ss_patgf_prob_dprs_p	Paternal grandfather depression problem	fam_history_q6b_depression	Paternal grandfather	Parent	baseline
	famhx_ss_patgm_prob_dprs_p	Paternal grandmother depression problem	fam_history_q6c_depression	Paternal grandmother	Parent	baseline
	famhx_ss_moth_prob_dprs_p	Mother depression problem	fam_history_q6d_depression	Biological mother	Parent	baseline

	famhx_ss_matgf_prob_dprs_p	Maternal grandfather depression problem		fam_history_q6e_depression	Maternal grandfather	Parent	baseline
	famhx_ss_matgm_prob_dprs_p	Maternal grandmother depression problem		fam_history_q6f_depression	Maternal grandmother	Parent	baseline
ABCD Parent Family History Summary Scores (abcd_fhxssp01)	famhx_ss_fath_prob_ma_p	Father mania problem	fhxp201	fam_history_7_yes_no	Has ANY blood relative of your child ever had a period of time when others were concerned because they suddenly became more active day and night and seemed not to need any sleep and talked much more than usual for them?	Parent	baseline
				fam_history_q7a_mania	Biological father	Parent	baseline
	famhx_ss_patgf_prob_ma_p	Paternal grandfather mania problem		fam_history_q7b_mania	Paternal grandfather	Parent	baseline
	famhx_ss_patgm_prob_ma_p	Paternal grandmother mania problem		fam_history_q7c_mania	Paternal grandmother	Parent	baseline
	famhx_ss_moth_prob_ma_p	Mother mania problem		fam_history_q7d_mania	Biological mother	Parent	baseline
	famhx_ss_matgf_prob_ma_p	Maternal grandfather mania problem		fam_history_q7e_mania	Maternal grandfather	Parent	baseline
	famhx_ss_matgm_prob_ma_p	Maternal grandmother mania problem		fam_history_q7f_mania	Maternal grandmother	Parent	baseline
	famhx_ss_fath_prob_vs_p	Father visions of others spying/plotting problem		fam_history_8_yes_no	Has ANY blood relative of your child ever had a period lasting six months when they saw visions or heard voices or thought people	Parent	baseline

			were spying on them or plotting against them?		
		fam_history_q8a_visions	Biological father	Parent	baseline
famhx_ss_patgf_prob_vs_p	Paternal grandfather visions of others spying/plotting problem	fam_history_q8b_visions	Paternal grandfather	Parent	baseline
famhx_ss_patgm_prob_vs_p	Paternal grandmother visions of others spying/plotting problem	fam_history_q8c_visions	Paternal grandmother	Parent	baseline
famhx_ss_moth_prob_vs_p	Mother visions of others spying/plotting problem	fam_history_q8d_visions	Biological mother	Parent	baseline
famhx_ss_matgf_prob_vs_p	Maternal grandfather visions of others spying/plotting problem	fam_history_q8e_visions	Maternal grandfather	Parent	baseline
famhx_ss_matgm_prob_vs_p	Maternal grandmother visions of others spying/plotting problem	fam_history_q8f_visions	Maternal grandmother	Parent	baseline
famhx_ss_fath_prob_trb_p	Father trouble holds job/fights/police problem	fam_history_9_yes_no	Has ANY blood relative of your child been the kind of person who never holds a job for long, or gets into fights, or gets into trouble with the police from time to time, or had any trouble with the law as a child or an adult?	Parent	baseline
		fam_history_q9a_trouble	Biological father	Parent	baseline
famhx_ss_patgf_prob_trb_p	Paternal grandfather trouble holds job/fights/police problem	fam_history_q9b_trouble	Paternal grandfather	Parent	baseline
famhx_ss_patgm_prob_trb_p	Paternal grandmother trouble holds job/fights/police problem	fam_history_q9c_trouble	Paternal grandmother	Parent	baseline

famhx_ss_moth_prob_trb_p	Mother trouble holds job/fights/police problem	fam_history_q9d_trouble	Biological mother	Parent	baseline
famhx_ss_matgf_prob_trb_p	Maternal grandfather trouble holds job/fights/police problem	fam_history_q9e_trouble	Maternal grandfather	Parent	baseline
famhx_ss_matgm_prob_trb_p	Maternal grandmother trouble holds job/fights/police problem	fam_history_q9f_trouble	Maternal grandmother	Parent	baseline
famhx_ss_fath_prob_nrv_p	Father nerves/nervous breakdown problem	fam_history_10_yes_no	Has ANY blood relative of your child ever had any other problems with their nerves, or had a nervous breakdown?	Parent	baseline
		fam_history_q10a_nerves	Biological father	Parent	baseline
famhx_ss_patgf_prob_nrv_p	Paternal grandfather nerves/nervous breakdown problem	fam_history_q10b_nerves	Paternal grandfather	Parent	baseline
famhx_ss_patgm_prob_nrv_p	Paternal grandmother nerves/nervous breakdown problem	fam_history_q10c_nerves	Paternal grandmother	Parent	baseline
famhx_ss_moth_prob_nrv_p	Mother nerves/nervous breakdown problem	fam_history_q10d_nerves	Biological mother	Parent	baseline
famhx_ss_matgf_prob_nrv_p	Maternal grandfather nerves/nervous breakdown problem	fam_history_q10e_nerves	Maternal grandfather	Parent	baseline
famhx_ss_matgm_prob_nrv_p	Maternal grandmother nerves/nervous breakdown problem	fam_history_q10f_nerves	Maternal grandmother	Parent	baseline
famhx_ss_fath_prob_prf_p	Father been to a doctor or counselor due to emotional/mental problem	fam_history_11_yes_no	Has ANY blood relative of your child ever been to a doctor or a counselor about any emotional or mental problems, or problems with alcohol or drugs?	Parent	baseline

ABCD Parent Family History Summary Scores (abcd_fhxssp01)	famhx_ss_fath_prob_prf_p	Father been to a doctor or counselor due to emotional/mental problem	fhxp201	fam_history_q11a_professional	Biological father	Parent	baseline
	famhx_ss_patgf_prob_prf_p	Paternal grandfather been to a doctor or counselor due to emotional/mental problem		fam_history_q11b_professional	Paternal grandfather	Parent	baseline
	famhx_ss_patgm_prob_prf_p	Paternal grandmother been to a doctor or counselor due to emotional/mental problem		fam_history_q11c_professional	Paternal grandmother	Parent	baseline
	famhx_ss_moth_prob_prf_p	Mother been to a doctor or counselor due to emotional/mental problem		fam_history_q11d_professional	Biological mother	Parent	baseline
	famhx_ss_matgf_prob_prf_p	Maternal grandfather been to a doctor or counselor due to emotional/mental problem		fam_history_q11e_professional	Maternal grandfather	Parent	baseline
	famhx_ss_matgm_prob_prf_p	Maternal grandmother been to a doctor or counselor due to emotional/mental problem		fam_history_q11f_professional	Maternal grandmother	Parent	baseline
	famhx_ss_fath_prob_hspd_p	Father hospitalized due to emotional/mental problem		fam_history_12_yes_no	Has ANY blood relative of your child ever been hospitalized because of emotional or mental problems, or drug or alcohol problems?	Parent	baseline
				fam_history_q12a_hospitalized	Biological father	Parent	baseline
	famhx_ss_patgf_prob_hspd_p	Paternal grandfather hospitalized due to emotional/mental problem		fam_history_q12b_hospitalized	Paternal grandfather	Parent	baseline
	famhx_ss_patgm_prob_hspd_p	Paternal grandmother hospitalized due to emotional/mental problem		fam_history_q12c_hospitalized	Paternal grandmother	Parent	baseline

	famhx_ss_moth_prob_hspd_p	Mother hospitalized due to emotional/mental problem	fam_history_q12d_hospitalized	Biological mother	Parent	baseline
	famhx_ss_matgf_prob_hspd_p	Maternal grandfather hospitalized due to emotional/mental problem	fam_history_q12e_hospitalized	Maternal grandfather	Parent	baseline
	famhx_ss_matgm_prob_hspd_p	Maternal grandmother hospitalized due to emotional/mental problem	fam_history_q12f_hospitalized	Maternal grandmother	Parent	baseline
	famhx_ss_fath_prob_scd_p	Father attempted or committed suicide	fam_history_13_yes_no	Has ANY blood relative of your child ever attempted or committed suicide?	Parent	baseline
			fam_history_q13a_suicide	Biological father	Parent	baseline
	famhx_ss_patgf_prob_scd_p	Paternal grandfather attempted or committed suicide	fam_history_q13b_suicide	Paternal grandfather	Parent	baseline
	famhx_ss_patgm_prob_scd_p	Paternal grandmother attempted or committed suicide	fam_history_q13c_suicide	Paternal grandmother	Parent	baseline
	famhx_ss_moth_prob_scd_p	Mother attempted or committed suicide	fam_history_q13d_suicide	Biological mother	Parent	baseline
	famhx_ss_matgf_prob_scd_p	Maternal grandfather attempted or committed suicide	fam_history_q13e_suicide	Maternal grandfather	Parent	baseline
	famhx_ss_matgm_prob_scd_p	Maternal grandmother attempted or committed suicide	fam_history_q13f_suicide	Maternal grandmother	Parent	baseline
ABCD Developmental History Questionnaire (dhx01)	devhx_3_p	How old were you/biological mother when the child was born?			Parent	baseline
	devhx_4_p	How old was the child's biological father when he/she was born?			Parent	baseline
	devhx_5_p	Does your child have a twin?			Parent	baseline

	devhx_6_p	Was your pregnancy with this child a planned pregnancy?	Parent	baseline
	devhx_7_p	How far along (in weeks) were you with your child when you found out that you were pregnant?	Parent	baseline
	devhx_8_prescript_med	Before the biological mother/you found out she was pregnant, but while she/you might have been pregnant with this child, did she/you use prescription medications?	Parent	baseline
ABCD Developmental History Questionnaire (dhx01)	devhx_8_tobacco	Before the biological mother/you found out she was pregnant, but while she/you might have been pregnant with this child, did she/you use tobacco?	Parent	baseline
	devhx_8_alcohol	Before the biological mother/you found out she was pregnant, but while she/you might have been pregnant with this child, did she/you use alcohol?	Parent	baseline
	devhx_8_marijuana	Before the biological mother/you found out she was pregnant, but while she/you might have been pregnant with this child, did she/you use marijuana?	Parent	baseline
	devhx_8_coc_crack	Before the biological mother/you found out she was pregnant, but while she/you might have been pregnant with this child, did she/you use cocaine/crack?	Parent	baseline

	devhx_8_her_morph	Before the biological mother/you found out she was pregnant, but while she/you might have been pregnant with this child, did she/you use heroin/morphine?		Parent	baseline
	devhx_8_oxycont	Before the biological mother/you found out she was pregnant, but while she/you might have been pregnant with this child, did she/you use oxycontin?		Parent	baseline
	devhx_8_other_drugs	Before the biological mother/you found out she was pregnant, but while she/you might have been pregnant with this child, did she/you use any other drugs?		Parent	baseline
	devhx_9_prescript_med	Once the biological mother/you knew she was pregnant, was she/you using prescription medications?		Parent	baseline
	devhx_9_tobacco	Once the biological mother/you knew she was pregnant, was she/you using tobacco?		Parent	baseline
	devhx_9_alcohol	Once the biological mother/you knew she was pregnant, was she/you using alcohol?		Parent	baseline
	devhx_9_marijuana	Once the biological mother/you knew she was pregnant, was she/you using marijuana?		Parent	baseline
	devhx_9_coc_crack	Once the biological mother/you knew she was pregnant, was she/you using cocaine/crack?		Parent	baseline

	devhx_9_her_morph	Once the biological mother/you knew she was pregnant, was she/you using heroin/morphine?		Parent	baseline
	devhx_9_oxycont	Once the biological mother/you knew she was pregnant, was she/you using oxycontin?		Parent	baseline
	devhx_9_other_drugs	Once the biological mother/you knew she was pregnant, was she/you using any other drugs?		Parent	baseline
	devhx_10	Did the biological mother/you take prenatal vitamins during the pregnancy?		Parent	baseline
	devhx_caffeine_11	Did the biological mother/you have any caffeine during pregnancy (from conception until delivery)?		Parent	baseline
	devhx_10a3_p	During the pregnancy with this child, did the biological mother/you have severe nausea and vomiting extending past the 6th month or accompanied by weight loss?		Parent	baseline
ABCD Developmental History Questionnaire (dhx01)	devhx_10b3_p	During the pregnancy with this child, did the biological mother/you have heavy bleeding requiring bed rest or special treatment?		Parent	baseline
	devhx_10c3_p	During the pregnancy with this child, did the biological mother/you have pre-eclampsia, eclampsia, or toxemia?		Parent	baseline
	devhx_10d3_p	During the pregnancy with this child, did the biological mother/you have a severe gall bladder attack?		Parent	baseline

	devhx_10e3_p	During the pregnancy with this child, did the biological mother/you have persistent proteinuria?		Parent	baseline
	devhx_10f3_p	During the pregnancy with this child, did the biological mother/you have rubella (German measles) during the first 3 months of pregnancy?		Parent	baseline
	devhx_10g3_p	During the pregnancy with this child, did the biological mother/you have severe anemia?		Parent	baseline
	devhx_10h3_p	During the pregnancy with this child, did the biological mother/you have urinary tract infections?		Parent	baseline
	devhx_10i3_p	During the pregnancy with this child, did the biological mother/you have pregnancy-related diabetes?		Parent	baseline
	devhx_10j3_p	During the pregnancy with this child, did the biological mother/you have pregnancy-related high blood pressure?		Parent	baseline
	devhx_10k3_p	During the pregnancy with this child, did the biological mother/you have previa, abruptio, or other problems with the placenta?		Parent	baseline
	devhx_10l3_p	During the pregnancy with this child, did the biological mother/you have an accident or injury requiring medical care?		Parent	baseline
	devhx_10m3_p	During the pregnancy with this child, did the biological mother/you have		Parent	baseline

	any other conditions requiring medical care?		
devhx_11_p	About how many times did you/biological mother see a doctor or other medical professional for prenatal care during this pregnancy?	Parent	baseline
devhx_12a_p	Was the child born prematurely?	Parent	baseline
devhx_13_3_p	Was the child born by Caesarian section?	Parent	baseline
devhx_14a3_p	Was the child blue at birth?	Parent	baseline
devhx_14b3_p	Did the child have a slow heart beat at birth?	Parent	baseline
devhx_14c3_p	Did the child not breathe at first at birth?	Parent	baseline
devhx_14d3_p	Did the child have convulsions at birth?	Parent	baseline
devhx_14e3_p	Did the child have jaundice needing treatment at birth?	Parent	baseline
devhx_14f3_p	Did the child require oxygen at birth?	Parent	baseline
devhx_14g3_p	Did the child require a blood transfusion at birth?	Parent	baseline
devhx_14h3_p	Did the child have Rh incompatibility at birth?	Parent	baseline
devhx_15	For how many days after birth was the child in an incubator?	Parent	baseline

	devhx_16_p	About how many days in the first 12 months of life, did the child have a fever of 104 degrees or greater?	Parent	baseline
ABCD Developmental History Questionnaire (dhx01)	devhx_17_p	About how many days in the first 12 months of life did the child have any infections or serious illnesses?	Parent	baseline
	devhx_18_p	For how many months was the child breast fed?	Parent	baseline
	devhx_19a_p	At approximately what age was the child FIRST able to roll over?	Parent	baseline
	devhx_19b_p	At approximately what age was the child FIRST able to sit without assistance?	Parent	baseline
	devhx_19c_p	At approximately what age was the child FIRST able to walk without assistance?	Parent	baseline
	devhx_19d_p	At approximately what age was the child FIRST able to say his/her first word?	Parent	baseline
	devhx_20_p	Would you say the child's motor development (sitting, crawling, walking) was earlier, average, or later than most other children?	Parent	baseline
	devhx_21_p	Would you say the child's speech development was earlier, average, or later than most other children?	Parent	baseline
	devhx_22_3_p	Has the child ever wet the bed at night?	Parent	baseline

ABCD Parent Diagnostic Interview for DSM-5 (KSADS) Traumatic Events (abcd_ptsd01)	ksads_ptsd_raw_754_p	A car accident in which your child or another person in the car was hurt bad enough to require medical attention	Parent	1-year follow-up
	ksads_ptsd_raw_755_p	Another significant accident for which your child needed specialized and intensive medical treatment	Parent	1-year follow-up
	ksads_ptsd_raw_756_p	Witnessed or caught in a fire that caused significant property damage or personal injury	Parent	1-year follow-up
	ksads_ptsd_raw_757_p	Witnessed or caught in a natural disaster that caused significant property damage or personal injury	Parent	1-year follow-up
	ksads_ptsd_raw_758_p	Witnessed or present during an act of terrorism (e.g., Boston marathon bombing)	Parent	1-year follow-up
	ksads_ptsd_raw_759_p	Witnessed death or mass destruction in a war zone	Parent	1-year follow-up
	ksads_ptsd_raw_760_p	Witnessed someone shot or stabbed in the community	Parent	1-year follow-up
	ksads_ptsd_raw_761_p	Shot, stabbed, or beaten brutally by a non-family member	Parent	1-year follow-up
	ksads_ptsd_raw_762_p	Shot, stabbed, or beaten brutally by a grown up in the home	Parent	1-year follow-up

	ksads_ptsd_raw_763_p	Beaten to the point of having bruises by a grown up in the home	Parent	1-year follow-up
	ksads_ptsd_raw_764_p	A non-family member threatened to kill your child	Parent	1-year follow-up
	ksads_ptsd_raw_765_p	A family member threatened to kill your child	Parent	1-year follow-up
	ksads_ptsd_raw_766_p	Witness the grownups in the home push, shove or hit one another	Parent	1-year follow-up
	ksads_ptsd_raw_767_p	A grown up in the home touched your child in his or her privates, had your child touch their privates, or did other sexual things to your child	Parent	1-year follow-up
ABCD Parent Diagnostic Interview for DSM-5 (KSADS) Traumatic Events (abcd_ptsd01)	ksads_ptsd_raw_768_p	An adult outside your family touched your child in his or her privates, had your child touch their privates or did other sexual things to your child	Parent	1-year follow-up
	ksads_ptsd_raw_769_p	A peer forced your child to do something sexually	Parent	1-year follow-up
	ksads_ptsd_raw_770_p	Learned about the sudden unexpected death of a loved one	Parent	1-year follow-up
Residential History Derived	reshist_addr1_valid	Residential history validity	Address - derived	baseline

Scores (abcd_rhds01)	reshist_addr1_status	Residential history status message	Address - derived	baseline
	reshist_addr1_years	Residential history years of residence	Address - derived	baseline
	reshist_addr1_d1a	Gross residential density	Address - derived	baseline
	reshist_addr1_walkindex	National walkability index	Address - derived	baseline
	reshist_addr1_grndtot	Uniform Crime Reports: grand total crimes	Address - derived	baseline
	reshist_addr1_p1tot	Uniform Crime Reports: total adult offenses	Address - derived	baseline
	reshist_addr1_p1vlnt	Uniform Crime Reports: adult violent crimes	Address - derived	baseline
	reshist_addr1_drugtot	Uniform Crime Reports: drug abuse violations total	Address - derived	baseline
	reshist_addr1_drgsale	Uniform Crime Reports: drug sale total	Address - derived	baseline

reshist_addr1_mjsale	Uniform Crime Reports: marijuana sale total	Address - derived	baseline
reshist_addr1_drgposs	Uniform Crime Reports: drug possession total	Address - derived	baseline
reshist_addr1_dui	Uniform Crime Reports: DUI	Address - derived	baseline
reshist_addr1_adi_edu_l	Area Deprivation Index: percentage of population aged at least 25 with less than 9 years of education	Address - derived	baseline
reshist_addr1_adi_edu_h	Area Deprivation Index: percentage of population aged at least 25 with at least a high school diploma	Address - derived	baseline
reshist_addr1_adi_work_c	Area Deprivation Index: percentage of employed persons aged at least 16 in white collar occupations	Address - derived	baseline
reshist_addr1_adi_income	Area Deprivation Index: median family income	Address - derived	baseline
reshist_addr1_adi_in_dis	Area Deprivation Index: income disparity (defined by Singh as the log of 100 x ratio of the number of households with <10,000 annual income to the number of households with >50,000 annual income)	Address - derived	baseline
reshist_addr1_adi_home_v	Area Deprivation Index: median home value	Address - derived	baseline

	reshist_addr1_adi_rent	Area Deprivation Index: median gross rent	Address - derived	baseline
	reshist_addr1_adi_mortg	Area Deprivation Index: median monthly mortgage	Address - derived	baseline
Residential History Derived Scores (abcd_rhds01)	reshist_addr1_adi_home_o	Area Deprivation Index: percentage of house/residence ownership	Address - derived	baseline
	reshist_addr1_adi_crowd	Area Deprivation Index: percentage of occupied housing units with more than 1 person per room (crowding)	Address - derived	baseline
	reshist_addr1_adi_unemp	Area Deprivation Index: percentage of civilian labor force population aged at least 16 unemployed (unemployment rate)	Address - derived	baseline
	reshist_addr1_adi_pov	Area Deprivation Index: percentage of families below the poverty level	Address - derived	baseline
	reshist_addr1_adi_b138	Area Deprivation Index: percentage of population below 138% of the poverty threshold	Address - derived	baseline
	reshist_addr1_adi_sp	Area Deprivation Index: percentage of single households	Address - derived	baseline
	reshist_addr1_adi_ncar	Area Deprivation Index: percentage of occupied housing units without a motor vehicle	Address - derived	baseline

reshist_addr1_adi_ntel	Area Deprivation Index: percentage of occupied housing units without a telephone	Address - derived	baseline
reshist_addr1_adi_nplumb	Area Deprivation Index: percentage of occupied housing units without complete plumbing	Address - derived	baseline
reshist_addr1_adi_wsum	Area Deprivation Index: scaled weighted sum (based on Kind et al., Annals of Internal Medicine, 2014)	Address - derived	baseline
reshist_addr1_adi_perc	Area Deprivation Index: national percentiles (higher means higher value of ADI)	Address - derived	baseline
reshist_addr1_popdensity	UN adjusted population density	Address - derived	baseline
reshist_addr1_no2	3 years average of ground level NO2 at 10x10km2	Address - derived	baseline
reshist_addr1_pm25	Annual average of PM 2.5 at 10x10km2	Address - derived	baseline
reshist_addr1_proxrd	Proximity to major roads (in meters)	Address - derived	baseline
reshist_addr1_percentile	Percentage of time spent at primary residential address	Address - derived	baseline

	reshist_addr1_pm252016aa	Annual average of PM 2.5 at 1x1km2	Address - derived	baseline
	reshist_addr1_leadrisk_poverty	Percentage of individuals below - 125% of the poverty threshold	Address - derived	baseline
	reshist_addr1_leadrisk_housing	Estimated percentage of homes at risk for lead exposure given lead-based paint	Address - derived	baseline
	reshist_addr1_leadrisk	Estimated lead risk (1-10 scale)	Address - derived	baseline
	reshist_state_sexism_factor	State level indicators of sexism from survey and implicit bias measures	Address - derived	baseline
	reshist_state_racism_factor	State level indicators of racism from survey and implicit bias measures and state level structural variables	Address - derived	baseline
	reshist_state_so_factor	State level indicators of bias against sexual orientation from structural variables	Address - derived	baseline
	reshist_state_immigrant_factor	State level indicators of immigrant bias from survey and implicit bias measures and state level structural variables	Address - derived	baseline
Residential History Derived Scores	reshist_state_mj_laws	Marijuana state law during the same year as the assessment	Address - derived	baseline

(abcd_rhds01<br>)				
	ple_died_y	Someone in family died	Youth	1-year follow-up
	ple_injured_y	Family member was seriously injured	Youth	1-year follow-up
	ple_crime_y	Saw crime or accident	Youth	1-year follow-up
	ple_friend_y	Lost a close friend	Youth	1-year follow-up
ABCD Youth Life Events (abcd_yle01)	ple_friend_injur_y	Close friend was seriously sick/injured	Youth	1-year follow-up
	ple_financial_y	Negative change in parent's financial situation	Youth	1-year follow-up
	ple_sud_y	Family member had drug and/or alcohol problem	Youth	1-year follow-up
	ple_ill_y	You got seriously sick	Youth	1-year follow-up
	ple_injur_y	You got seriously injured	Youth	1-year follow-up

	ple_argue_y	Parents argued more than previously	Youth	1-year follow-up
	ple_job_y	Mother/father figure lost job	Youth	1-year follow-up
	ple_away_y	One parent was away from home more often	Youth	1-year follow-up
	ple_arrest_y	Someone in the family was arrested	Youth	1-year follow-up
	ple_friend_died_y	Close friend died	Youth	1-year follow-up
	ple_mh_y	Family member had mental/emotional problem	Youth	1-year follow-up
	ple_sib_y	Brother or sister left home	Youth	1-year follow-up
	ple_victim_y	Was a victim of crime/violence/assault	Youth	1-year follow-up
	ple_separ_y	Parents separated or divorced	Youth	1-year follow-up

	ple_law_y	Parents/caregiver got into trouble with the law				Youth	1-year follow-up
	ple_school_y	Attended a new school				Youth	1-year follow-up
	ple_move_y	Family moved				Youth	1-year follow-up
	ple_jail_y	One of the parents/caregivers went to jail				Youth	1-year follow-up
	ple_step_y	Got new stepmother or stepfather				Youth	1-year follow-up
	ple_new_job_y	Parent/caregiver got a new job				Youth	1-year follow-up
	ple_new_sib_y	Got new brother or sister				Youth	1-year follow-up

Sum Scores Mental Health Youth (abcd_mhy02)	ple_y_ss_total_number	Total number of events	abcd_yle01	ple_died_y	Someone in family died	Youth	1-year follow-up
				ple_injured_y	Family member was seriously injured	Youth	1-year follow-up

	ple_crime_y	Saw crime or accident	Youth	1-year follow-up
	ple_friend_y	Lost a close friend	Youth	1-year follow-up
	ple_friend_injur_y	Close friend was seriously sick/injured	Youth	1-year follow-up
	ple_financial_y	Negative change in parent's financial situation	Youth	1-year follow-up
	ple_sud_y	Family member had drug and/or alcohol problem	Youth	1-year follow-up
	ple_ill_y	You got seriously sick	Youth	1-year follow-up
	ple_injur_y	You got seriously injured	Youth	1-year follow-up
	ple_argue_y	Parents argued more than previously	Youth	1-year follow-up
	ple_job_y	Mother/father figure lost job	Youth	1-year follow-up

	ple_away_y	One parent was away from home more often	Youth	1-year follow-up
	ple_arrest_y	Someone in the family was arrested	Youth	1-year follow-up
	ple_friend_died_y	Close friend died	Youth	1-year follow-up
	ple_mh_y	Family member had mental/emotional problem	Youth	1-year follow-up
	ple_sib_y	Brother or sister left home	Youth	1-year follow-up
	ple_victim_y	Was a victim of crime/violence/assault	Youth	1-year follow-up
	ple_separ_y	Parents separated or divorced	Youth	1-year follow-up
	ple_law_y	Parents/caregiver got into trouble with the law	Youth	1-year follow-up
	ple_school_y	Attended a new school	Youth	1-year follow-up

				ple_move_y	Family moved	Youth	1-year follow-up
				ple_jail_y	One of the parents/caregivers went to jail	Youth	1-year follow-up
				ple_step_y	Got new stepmother or stepfather	Youth	1-year follow-up
				ple_new_job_y	Parent/caregiver got a new job	Youth	1-year follow-up
				ple_new_sib_y	Got new brother or sister	Youth	1-year follow-up
	ple_y_ss_total_good	Total number of good events		ple_died_fu_y	Was this a good or bad experience?	Youth	1-year follow-up
				ple_injured_fu_y	Was this a good or bad experience?	Youth	1-year follow-up
				ple_crime_fu_y	Was this a good or bad experience?	Youth	1-year follow-up
Sum Scores Mental Health Youth	ple_y_ss_total_good	Total number of good events	abcd_yle01	ple_friend_fu_y	Was this a good or bad experience?	Youth	1-year follow-up

(abcd_mhy02<br>)		ple_friend_injur_fu_y	Was this a good or bad experience?	Youth	1-year follow-up
		ple_financial_fu_y	Was this a good or bad experience?	Youth	1-year follow-up
		ple_sud_fu_y	Was this a good or bad experience?	Youth	1-year follow-up
		ple_ill_fu_y	Was this a good or bad experience?	Youth	1-year follow-up
		ple_injur_fu_y	Was this a good or bad experience?	Youth	1-year follow-up
		ple_argue_fu_y	Was this a good or bad experience?	Youth	1-year follow-up
		ple_job_fu_y	Was this a good or bad experience?	Youth	1-year follow-up
		ple_away_fu_y	Was this a good or bad experience?	Youth	1-year follow-up
		ple_arrest_fu_y	Was this a good or bad experience?	Youth	1-year follow-up

		ple_friend_died_fu_y	Was this a good or bad experience?	Youth	1-year follow-up
		ple_mh_fu_y	Was this a good or bad experience?	Youth	1-year follow-up
		ple_sib_fu_y	Was this a good or bad experience?	Youth	1-year follow-up
		ple_victim_fu_y	Was this a good or bad experience?	Youth	1-year follow-up
ple_y_ss_total_bad	Total number of bad events	ple_separ_fu_y	Was this a good or bad experience?	Youth	1-year follow-up
		ple_law_fu_y	Was this a good or bad experience?	Youth	1-year follow-up
		ple_school_fu_y	Was this a good or bad experience?	Youth	1-year follow-up
		ple_move_fu_y	Was this a good or bad experience?	Youth	1-year follow-up
		ple_jail_fu_y	Was this a good or bad experience?	Youth	1-year follow-up

		ple_step_fu_y	Was this a good or bad experience?	Youth	1-year follow-up
		ple_new_job_fu_y	Was this a good or bad experience?	Youth	1-year follow-up
		ple_new_sib_fu_y	Was this a good or bad experience?	Youth	1-year follow-up
ple_y_ss_affect_sum	How much affected (sum)	ple_died_fu2_y	How much did the event affect you?	Youth	1-year follow-up
		ple_injured_fu2_y	How much did the event affect you?	Youth	1-year follow-up
		ple_crime_fu2_y	How much did the event affect you?	Youth	1-year follow-up
		ple_friend_fu2_y	How much did the event affect you?	Youth	1-year follow-up
		ple_friend_injur_fu2_y	How much did the event affect you?	Youth	1-year follow-up
		ple_financial_fu2_y	How much did the event affect you?	Youth	1-year follow-up

Sum Scores Mental Health Youth (abcd_mhy02)	ple_y_ss_affect_sum	How much affected (sum)	abcd_yle01	ple_sud_fu2_y	How much did the event affect you?	Youth	1-year follow-up
	ple_y_ss_affected_good_sum	How much affected (good) (sum)		ple_ill_fu2_y	How much did the event affect you?	Youth	1-year follow-up
				ple_injur_fu2_y	How much did the event affect you?	Youth	1-year follow-up
				ple_argue_fu2_y	How much did the event affect you?	Youth	1-year follow-up
				ple_job_fu2_y	How much did the event affect you?	Youth	1-year follow-up
				ple_away_fu2_y	How much did the event affect you?	Youth	1-year follow-up
				ple_arrest_fu2_y	How much did the event affect you?	Youth	1-year follow-up
	ple_y_ss_affected_bad_sum	How much affected (bad) (sum)		ple_friend_died_fu2_y	How much did the event affect you?	Youth	1-year follow-up
				ple_mh_fu2_y	How much did the event affect you?	Youth	1-year follow-up

		ple_sib_fu2_y	How much did the event affect you?	Youth	1-year follow-up
		ple_victim_fu2_y	How much did the event affect you?	Youth	1-year follow-up
		ple_separ_fu2_y	How much did the event affect you?	Youth	1-year follow-up
		ple_law_fu2_y	How much did the event affect you?	Youth	1-year follow-up
	ple_y_ss_affected_bad_mean	How much affected (bad) (mean)	ple_school_fu2_y	How much did the event affect you?	Youth	1-year follow-up
			ple_move_fu2_y	How much did the event affect you?	Youth	1-year follow-up
			ple_jail_fu2_y	How much did the event affect you?	Youth	1-year follow-up
			ple_step_fu2_y	How much did the event affect you?	Youth	1-year follow-up
			ple_new_job_fu2_y	How much did the event affect you?	Youth	1-year follow-up

				ple_new_sib_fu2_y	How much did the event affect you?	Youth	1-year follow-up
Sum Scores Mental Health Parent (abcd_mhp02)	ple_p_ss_total_number	Total number of events	abcd_ple01	ple_died_p	Someone in family died	Parent	1-year follow-up
				ple_injured_p	Family member was seriously injured	Parent	1-year follow-up
				ple_crime_p	Saw crime or accident	Parent	1-year follow-up
				ple_friend_p	Lost a close friend	Parent	1-year follow-up
				ple_friend_injur_p	Close friend was seriously sick/injured	Parent	1-year follow-up
				ple_financial_p	Negative change in parent's financial situation	Parent	1-year follow-up
				ple_sud_p	Family member had drug and/or alcohol problem	Parent	1-year follow-up
				ple_ill_p	You got seriously sick	Parent	1-year follow-up

				ple_injur_p	You got seriously injured	Parent	1-year follow-up
Sum Scores Mental Health Parent (abcd_mhp02)	ple_p_ss_total_number	Total number of events	abcd_ple01	ple_argue_p	Parents argued more than previously	Parent	1-year follow-up
				ple_job_p	Mother/father figure lost job	Parent	1-year follow-up
				ple_away_p	One parent was away from home more often	Parent	1-year follow-up
				ple_arrest_p	Someone in the family was arrested	Parent	1-year follow-up
				ple_friend_died_p	Close friend died	Parent	1-year follow-up
				ple_mh_p	Family member had mental/emotional problem	Parent	1-year follow-up
				ple_sib_p	Brother or sister left home	Parent	1-year follow-up
				ple_victim_p	Was a victim of crime/violence/assault	Parent	1-year follow-up

		ple_separ_p	Parents separated or divorced	Parent	1-year follow-up
		ple_law_p	Parents/caregiver got into trouble with the law	Parent	1-year follow-up
		ple_school_p	Attended a new school	Parent	1-year follow-up
		ple_move_p	Family moved	Parent	1-year follow-up
		ple_jail_p	One of the parents/caregivers went to jail	Parent	1-year follow-up
		ple_step_p	Got new stepmother or stepfather	Parent	1-year follow-up
		ple_new_job_p	Parent/caregiver got a new job	Parent	1-year follow-up
		ple_new_sib_p	Got new brother or sister	Parent	1-year follow-up
ple_p_ss_total_good	Total number of good events	ple_died_fu_p	Was this a good or bad experience for your child?	Parent	1-year follow-up

		ple_injured_fu_p	Was this a good or bad experience for your child?	Parent	1-year follow-up
		ple_crime_fu_p	Was this a good or bad experience for your child?	Parent	1-year follow-up
		ple_friend_fu_p	Was this a good or bad experience for your child?	Parent	1-year follow-up
		ple_friend_injur_fu_p	Was this a good or bad experience for your child?	Parent	1-year follow-up
		ple_financial_fu_p	Was this a good or bad experience for your child?	Parent	1-year follow-up
		ple_sud_fu_p	Was this a good or bad experience for your child?	Parent	1-year follow-up
		ple_ill_fu_p	Was this a good or bad experience for your child?	Parent	1-year follow-up
		ple_injur_fu_p	Was this a good or bad experience for your child?	Parent	1-year follow-up
		ple_argue_fu_p	Was this a good or bad experience for your child?	Parent	1-year follow-up

				ple_job_fu_p	Was this a good or bad experience for your child?	Parent	1-year follow-up
				ple_away_fu_p	Was this a good or bad experience for your child?	Parent	1-year follow-up
Sum Scores Mental Health Parent (abcd_mhp02)	ple_p_ss_total_good	Total number of good events	abcd_ple01	ple_arrest_fu_p	Was this a good or bad experience for your child?	Parent	1-year follow-up
	ple_p_ss_total_bad	Total number of bad events		ple_friend_died_fu_p	Was this a good or bad experience for your child?	Parent	1-year follow-up
				ple_mh_fu_p	Was this a good or bad experience for your child?	Parent	1-year follow-up
				ple_sib_fu_p	Was this a good or bad experience for your child?	Parent	1-year follow-up
				ple_victim_fu_p	Was this a good or bad experience for your child?	Parent	1-year follow-up
				ple_separ_fu_p	Was this a good or bad experience for your child?	Parent	1-year follow-up
				ple_law_fu_p	Was this a good or bad experience for your child?	Parent	1-year follow-up

		ple_school_fu_p	Was this a good or bad experience for your child?	Parent	1-year follow-up
		ple_move_fu_p	Was this a good or bad experience for your child?	Parent	1-year follow-up
		ple_jail_fu_p	Was this a good or bad experience for your child?	Parent	1-year follow-up
		ple_step_fu_p	Was this a good or bad experience for your child?	Parent	1-year follow-up
		ple_new_job_fu_p	Was this a good or bad experience for your child?	Parent	1-year follow-up
		ple_new_sib_fu_p	Was this a good or bad experience for your child?	Parent	1-year follow-up
		ple_died_fu2_p	How much did the event affect your child?	Parent	1-year follow-up
	ple_p_ss_affected_good_sum	How much affected (good) (sum)	ple_injured_fu2_p	How much did the event affect your child?	Parent	1-year follow-up
		ple_crime_fu2_p	How much did the event affect your child?	Parent	1-year follow-up

		ple_friend_fu2_p	How much did the event affect your child?	Parent	1-year follow-up
		ple_friend_injur_fu2_p	How much did the event affect your child?	Parent	1-year follow-up
		ple_financial_fu2_p	How much did the event affect your child?	Parent	1-year follow-up
		ple_sud_fu2_p	How much did the event affect your child?	Parent	1-year follow-up
		ple_ill_fu2_p	How much did the event affect your child?	Parent	1-year follow-up
		ple_injur_fu2_p	How much did the event affect your child?	Parent	1-year follow-up
ple_p_ss_affected_bad_sum	How much affected (bad) (sum)	ple_argue_fu2_p	How much did the event affect your child?	Parent	1-year follow-up
		ple_job_fu2_p	How much did the event affect your child?	Parent	1-year follow-up
		ple_away_fu2_p	How much did the event affect your child?	Parent	1-year follow-up

				ple_arrest_fu2_p	How much did the event affect your child?	Parent	1-year follow-up
	ple_p_ss_affected_mean	How much affected (bad) (mean)		ple_friend_died_fu2_p	How much did the event affect your child?	Parent	1-year follow-up
				ple_mh_fu2_p	How much did the event affect your child?	Parent	1-year follow-up
Sum Scores Mental Health Parent (abcd_mhp02)	ple_p_ss_affected_mean	How much affected (bad) (mean)	abcd_ple01	ple_sib_fu2_p	How much did the event affect your child?	Parent	1-year follow-up
				ple_victim_fu2_p	How much did the event affect your child?	Parent	1-year follow-up
				ple_separ_fu2_p	How much did the event affect your child?	Parent	1-year follow-up
				ple_law_fu2_p	How much did the event affect your child?	Parent	1-year follow-up
	ple_p_ss_affect_sum	How much affected (sum)		ple_school_fu2_p	How much did the event affect your child?	Parent	1-year follow-up
				ple_move_fu2_p	How much did the event affect your child?	Parent	1-year follow-up

			ple_jail_fu2_p	How much did the event affect your child?	Parent	1-year follow-up
			ple_step_fu2_p	How much did the event affect your child?	Parent	1-year follow-up
			ple_new_job_fu2_p	How much did the event affect your child?	Parent	1-year follow-up
			ple_new_sib_fu2_p	How much did the event affect your child?	Parent	1-year follow-up
ABCD Longitudinal Parent Demographics Survey (abcd_lpds01)	demo_fam_exp1_v2_l	Needed food but couldn't afford to buy it or couldn't afford to go out to get it			Parent	1-year follow-up
	demo_fam_exp2_v2_l	Were without telephone service because you could not afford it			Parent	1-year follow-up
	demo_fam_exp3_v2_l	Didn't pay the full amount of the rent or mortgage because you could not afford it			Parent	1-year follow-up
	demo_fam_exp4_v2_l	Were evicted from your home for not paying the rent or mortgage			Parent	1-year follow-up
	demo_fam_exp5_v2_l	Had services turned off by the gas or electric company, or the oil company wouldn't deliver oil because payments were not made			Parent	1-year follow-up

	demo_fam_exp6_v2_l	Had someone who needed to see a doctor or go to the hospital but didn't go because you could not afford it	Parent	1-year follow-up
	demo_fam_exp7_v2_l	Had someone who needed a dentist but couldn't go because you could not afford it	Parent	1-year follow-up
	demo_yrs_1_l	How often does your child attend religious services?	Parent	1-year follow-up
	demo_yrs_2_l	In general, how important are your child's religious and spiritual beliefs in his/her daily life?	Parent	1-year follow-up

The full range of ABCD exposome variables used in factor analysis and generation. The ABCD instrument and item descriptions for each of the 348 measures that went directly into exploratory factor analyses are displayed in the leftmost three columns. Some of these measures were ABCD-derived summary variables; in these cases, the constituent instruments and items are described in the subsequent three columns, comprising the full 798 variables utilized in analysis. For every item, reporter and time point are displayed as well. Measures relating to genetics, neuroimaging, psychological state, and substance use were omitted.

Supplementary Table 2 Overview of ABCD instruments assessing mental health items

ABCD instrument	Amount of items	What	Reporter	Time point
ABCD Parent Diagnostic Interview for DSM-5 Full (KSADS-5) (abcd_ksad01)	18	Externalizing symptoms	Parent	1-year follow-up
ABCD Prodromal Psychosis Scale (pps01)	21	Psychosis	Youth	1-year follow-up
ABCD Youth Brief Problem Monitor (abcd_bpm01)	19	Internalizing symptoms	Youth	1-year follow-up
ABCD Youth NIH Toolbox Positive Affect Items (abcd_ytbpai01)	9	Positive affect	Youth	1-year follow-up
ABCD Youth Diagnostic Interview for DSM-5 (KSADS-5) (abcd_ksad501)	16	Suicidality	Youth	1-year follow-up
ABCD Parent General Behavior Inventory-Mania (PGBI) (abcd_pgbi01)	10	Mania	Parent	1-year follow-up

Supplementary Table 3 Missing data

Variable	Available data	Missing values
**Outcome**		
P-factor	11189	46
Psychosis factor	11189	46
**Exposome factors**		
Household adversity	11235	0
neighborhood environment	11235	0
day-to-day experiences	11235	0
state-level environment	11235	0
family values	11235	0
Pregnancy/birth complications	11235	0
**Covariates**		
Age	11235	0
Sex	11235	0
Race (white)	11235	0
Race (black)	11235	0
Ethnicity (hisp)	11098	137
Parent education	11177	58
Household income	10364	871

Supplementary Table 4. Correlated traits factor analysis of optimized collection of exposome variables

Item	F1	F2	F3	F4	F5	F6
Prenatal exposure to tobacco or marijuana	0.708					
Parental lifestyle issues (e.g., trouble with holding job, police, alcohol use)	0.701					
Physical conflict among adults at the home	0.653					
Inability to afford necessary medical/dental visit	0.593					
Severe family poverty (e.g., inability to afford necessities)	0.536	0.259				
Parental separation	0.536					
Prenatal exposure to hard drugs (e.g., cocaine, heroin)	0.535					
Planned pregnancy	-0.502	-0.205				
Enforced family rules for smoking cigarettes	-0.494					
Family legal trouble (e.g., arrests, jailtime)	0.456		-0.273			
Caregiver psychopathology (e.g., mood, personality, attention disorders)	0.416					
Severe maternal mental health issues (e.g., breakdowns, delusions, hospitalizations)	0.411					
Parent-reported sexual abuse	0.400					
Sudden death of a loved one	0.311					
Severe paternal mental health issues (e.g., breakdowns, delusions, hospitalizations)	0.291					
Prenatal exposure to alcohol	0.254	-0.221				
Parent-reported childhood trauma (e.g., accident, disaster, extreme violence)	0.156					

Census-derived neighborhood poverty (e.g., unemployment rate, families/individuals below poverty level)		0.845			
Parental ability to speak English	0.328	-0.671			
Census-derived neighborhood immigration and crowding		0.604			
Census-derived neighborhood population density		0.590			
Parent-reported neighborhood safety		-0.586			
Census-derived neighborhood lead exposure risk		0.580			-0.159
Census-derived neighborhood walkability index		0.429			
Census-derived neighborhood air pollution (NO2, PM25)		0.403			
Youth-reported acceptance and love by primary caregiver			0.572		
Youth-reported positive school involvement			0.570		
Youth-reported racial/ethnic discrimination (past year)			-0.568		
Youth-reported parental monitoring and communication			0.565		
Youth-reported school enjoyment			0.536		
Youth-reported lesbian, gay, bisexual discrimination (past year)			-0.514		
Youth-reported family conflict			-0.511		
Youth-reported discrimination based on weight (past year)			-0.504		
Youth-reported acceptance and love by secondary caregiver			0.486		
Youth-reported school grades and achievement			0.476		
Youth-reported unfair treatment on racial/ethnic grounds (lifetime)			-0.410		

Youth-reported positive feedback at school			0.409			
Youth-reported family discordance (e.g., loss of job, mental health issues, conflict/violence)			-0.399			
Youth-reported neighborhood safety		-0.333	0.361			
Youth-reported exposure to serious injury, illness, death (self or other)			-0.335			
Youth-reported discrimination based on being foreign (past year)		0.198	-0.313			
State-level indicators bias against sexual orientation		-0.284		0.939		
State-level indicators of sexism				0.791		
State-level marijuana laws				0.754		
State-level indicators of bias against immigrants		-0.270		0.724		
State-level indicators of racism				0.688		
State-level legality of medical marijuana				0.683		
Census-derived neighborhood wealth (e.g., median mortgage, rent, income)	-0.385			-0.434		
Family rules for using marijuana					0.945	
Family rules for smoking cigarettes					0.944	
Family rules for drinking alcohol					0.912	
Parent-reported importance of coherence to the family unit		0.265			0.490	
Parent-reported importance of family support		0.127			0.465	
Parent-reported importance of obligation to family		0.238			0.445	
Parent-reported importance of religion		0.180		0.220	0.405	
Family religiosity (e.g., attendance to religious services)				0.176	0.323	
Premature birth						0.846
Twin brother or sister						0.824

Blood oxygen complications at birth (e.g., jaundice, supplemental oxygen)	0.645
Time after birth in an incubator	0.497
Placental complications during pregnancy (e.g., previa, abruptio, persistent proteinuria)	0.473
Birth by caesarian section	0.469
Amount of prenatal care	0.414
Blood pressure complications during pregnancy (e.g., pregnancy-related high blood pressure, diabetes)	0.385
Circulation complications at birth (e.g., blue, slow heartbeat at birth)	0.335

Results of correlated traits factor analysis of the final set of exposome items, using iterated target rotation designed to detect complex structure (cross-loadings). *Factor 1* comprises variables most related to *household adversity*. *Factor 2* comprises variables most related to *neighborhood environment*. *Factor 3* comprises variables most related to youth-reported *day-to-day experiences*. *Factor 4* comprises variables most related to *state-level environment*. *Factor 5* comprises variables most related to *family values*. *Factor 6* includes variables most related to *pregnancy and birth complications*. Inter-factor correlations are shown at the bottom of the table. Columns B and C break down each item into its constituent ABCD variables. More information about full ABCD measures can be found in(11).

Supplementary Table 5 Associations of exposome factor scores with the PQ-B severity score

	PQ-B severity score (separate models)[a]			PQ-B severity score (single model)[b]		
**Exposome Factor**	**Beta**	**95% CI**	**P**	**Beta**	**95% CI**	**P**
Household Adversity	1.513	1.311–1.714	<0.001	1.898	1.660–2.136	<0.001
Neighborhood Environment	0.256	0.017–0.494	0.035	0.788	0.529–1.048	<0.001
Day-to-day Experiences	3.358	3.187–3.529	<0.001	3.681	3.491–3.871	<0.001
State-level Environment	0.192	0.010– 0.374	0.038	0.447	0.265– 0.629	<0.001
Family Values	-0.214	-0.393– -0.035	0.019	-0.054	-0.229– 0.121	0.548
Pregnancy/birth Complications	0.395	0.219– 0.571	<0.001	0.290	0.116– 0.463	0.001

[a] Each exposome factor was tested in a separate model (six models).

[b] All exposome factors are included in a single model. To address co-linearity among exposome factors, they were regressed out of each other.

Models co-varied for age, sex, race, ethnicity, household income, and parental education. Beta: standardized coefficient, CI: confidence interval, PQ-B: ABCD Prodromal Psychosis Scale

Supplementary Table 6 Explained variance (R^2) of the exposome factors and the covariates on the p-factor, psychosis factor, as well as the PQ-B

	General p-factor	Psychosis subdomain	PQ-B severity score
Only covariates	3.9%	3.9%	3.9%
Household Adversity	10.7%	3.9%	5.9%
Neighborhood Environment	4%	3.9%	4%
Day-to-day Experiences	39.4%	4.7%	16.1%
State-level Environment	4.4%	3.9%	4%
Family Values	4.2%	3.9%	4%
Pregnancy/birth Complications	4.1%	4%	4.1%

Each exposome factor was tested in a separate model (six models for each dependent variable). Beta: standardized coefficients, CI: confidence interval, covariates: age, sex, race, ethnicity, household income, and parental education, PQ-B: ABCD Prodromal Psychosis Scale

Supplementary Table 7 Associations of exposome factor scores with general p-factor imputed for missing demographic variables

	General p-factor (separate models)[a]			General p-factor (single model)[b]		
**Exposome Factor**	**Beta**	**95% CI**	**P**	**Beta**	**95% CI**	**P**
Household Adversity	0.309	0.288–0.329	<0.001	0.378	0.357–0.399	<0.001
Neighborhood Environment	0.028	0.004– 0.053	0.025	0.131	0.108– 0.154	<0.001
Day-to-day Experiences	0.621	0.606–0.636	<0.001	0.682	0.665–0.699	<0.001
State-level Environment	0.067	0.048–0.087	<0.001	0.118	0.102–0.134	<0.001
Family Values	-0.049	-0.067– -0.030	<0.001	-0.022	-0.037– -0.007	0.005
Pregnancy/birth Complications	0.035	0.016–0.053	<0.001	0.016	0.001–0.032	0.037

[a]Each exposome factor was tested in a separate model (six models).

[b]All exposome factors are included in a single model. To address co-linearity among exposome factors, they were regressed out of each other.

Models co-varied for age, sex, race, ethnicity, household income, and parental education. Beta: standardized coefficient, CI: confidence interval

Supplementary Table 8 Associations of exposome factor scores with psychosis factor imputed for missing demographic variables

	Psychosis subdomain (separate models)[a]			Psychosis subdomain (single model)[b]		
**Exposome Factor**	**Beta**	**95% CI**	**P**	**Beta**	**95% CI**	**P**
Household Adversity	0.021	-0.001–0.042	0.056	0.027	0.001–0.054	0.045
Neighborhood Environment	0.012	-0.012–0.037	0.327	0.022	-0.007– 0.051	0.143
Day-to-day Experiences	0.087	0.068–0.106	<0.001	0.093	0.072–0.114	<0.001
State-level Environment	-0.025	-0.044– -0.006	0.011	-0.020	-0.041–0.000	0.050
Family Values	0.000	-0.018–0.019	0.994	0.004	-0.015– 0.023	0.680
Pregnancy/birth Complications	0.033	0.015–0.052	<0.001	0.031	0.012–0.051	0.002

[a] Each exposome factor was tested in a separate model (six models).

[b] All exposome factors are included in a single model. To address co-linearity among exposome factors, they were regressed out of each other.

Models co-varied for age, sex, race, ethnicity, household income, and parental education. Beta: standardized coefficient, CI: confidence interval

Supplementary Table 9 Associations of exposome factor scores with general p-factor adjusted for family and site

	General p-factor (separate models)[a]			General p-factor (single model)[b]		
**Exposome Factor**	**Beta**	**95% CI**	**P**	**Beta**	**95% CI**	**P**
Household Adversity	0.313	0.291–0.335	<0.001	0.378	0.354–0.401	<0.001
Neighborhood Environment	0.042	0.013– 0.072	0.005	0.121	0.094– 0.147	<0.001
Day-to-day Experiences	0.619	0.603–0.634	<0.001	0.680	0.662–0.698	<0.001
State-level Environment	0.076	0.040–0.111	<0.001	0.112	0.080–0.145	<0.001
Family Values	-0.059	-0.079– -0.038	<0.001	-0.025	-0.043– -0.008	0.004
Pregnancy/birth Complications	0.044	0.021–0.066	<0.001	0.022	0.004–0.040	0.020

[a] Each exposome factor was tested in a separate model (six models).

[b] All exposome factors are included in a single model. To address co-linearity among exposome factors, they were regressed out of each other.

Models co-varied for age, sex, race, ethnicity, household income, and parental education. Beta: standardized coefficient, CI: confidence interval

Supplementary Table 10 Associations of exposome factor scores with psychosis factor adjusted for family and site

	Psychosis subdomain (separate models)[a]			Psychosis subdomain (single model)[b]		
**Exposome Factor**	**Beta**	**95% CI**	**P**	**Beta**	**95% CI**	**P**
Household Adversity	0.039	0.016–0.061	0.001	0.052	0.020–0.084	0.001
Neighborhood Environment	0.05	0.021–0.079	0.001	0.076	0.042– 0.111	<0.001
Day-to-day Experiences	0.097	0.077–0.116	<0.001	0.108	0.085–0.131	<0.001
State-level Environment	0.028	-0.045– 0.100	0.453	-0.041	-0.131–0.049	0.369
Family Values	0.004	-0.016–0.024	0.688	0.005	-0.019– 0.030	0.675
Pregnancy/birth Complications	-0.022	-0.045– -0.000	0.046	-0.037	-0.061– -0.013	0.003

[a] Each exposome factor was tested in a separate model (six models).

[b] All exposome factors are included in a single model. To address co-linearity among exposome factors, they were regressed out of each other.

Models co-varied for age, sex, race, ethnicity, household income, and parental education. Beta: standardized coefficient, CI: confidence interval

Supplementary References

1. Moore T, Martin I, Gur O, Jackson C, Scott J, Calkins M, et al. (2016): Characterizing social environment's association with neurocognition using census and crime data linked to the Philadelphia Neurodevelopmental Cohort. *Psychol Med*. 46:599-610.
2. Moore TM (2013): *Iteration of target matrices in exploratory factor analysis*. University of California, Los Angeles.
3. Moore TM, Reise SP, Depaoli S, Haviland MG (2015): Iteration of Partially Specified Target Matrices: Applications in Exploratory and Bayesian Confirmatory Factor Analysis. *Multivariate Behav Res*. 50:149-161.
4. Browne MW (1972): Oblique rotation to a partially specified target. *British Journal of Mathematical Statistical Psychology*. 25:207-212.
5. Harrington D (2009): *Confirmatory factor analysis*. Oxford university press.
6. Stekhoven DJ (2015): missForest: Nonparametric missing value imputation using random forest. *Astrophysics Source Code Library*.ascl: 1505.1011.
7. Caspi A, Moffitt TE (2018): All for one and one for all: Mental disorders in one dimension. *American Journal of Psychiatry*. 175:831-844.
8. Clark DA, Hicks BM, Angstadt M, Rutherford S, Taxali A, Hyde L, et al. (2021): The General Factor of Psychopathology in the Adolescent Brain Cognitive Development (ABCD) Study: A Comparison of Alternative Modeling Approaches. *Clin Psychol Sci*. 9:169-182.
9. Lahey BB, Krueger RF, Rathouz PJ, Waldman ID, Zald DH (2017): A hierarchical causal taxonomy of psychopathology across the life span. *Psychol Bull*. 143:142-186.
10. Moore TM, Lahey BB (2021): Issues in Estimating Interpretable Lower Order Factors in Second-Order Hierarchical Models: Commentary on Clark et al.(2021). *Clinical Psychological Science*.21677026211035114.
11. Moore TM, Visoki E, Argabright ST, DiDomenico GE, Sotelo I, Wortzel JD, et al. (2021): The exposome and its associations with broad mental and physical health measures in early adolescence. *medRxiv*.
